# Single-cell transcriptomics identifies an effectorness gradient shaping the response of CD4^+^ T cells to cytokines

**DOI:** 10.1038/s41467-020-15543-y

**Published:** 2020-04-14

**Authors:** Kiki Cano-Gamez, Blagoje Soskic, Theodoros I. Roumeliotis, Ernest So, Deborah J. Smyth, Marta Baldrighi, David Willé, Nikolina Nakic, Jorge Esparza-Gordillo, Christopher G. C. Larminie, Paola G. Bronson, David F. Tough, Wendy C. Rowan, Jyoti S. Choudhary, Gosia Trynka

**Affiliations:** 1 Wellcome Sanger Institute, Wellcome Genome Campus, Hinxton, CB10 1SA UK; 2 Open Targets, Wellcome Genome Campus, Cambridge, CB10 1SA UK; 3 Functional Proteomics, The Institute of Cancer Research, London, SW3 6JB UK; 4 Biostatistics, GSK R&D, Stevenage, SG1 2NY UK; 5 Functional Genomics, Medicinal Science and Technology, GSK R&D, Stevenage, SG1 2NY UK; 6 Human Genetics, GSK R&D, Stevenage, SG1 2NY UK; 7 Human Target Validation Core, R&D Translational Biology, Biogen, Cambridge, MA USA; 8 Adaptive Immunity RU, GSK R&D, Stevenage, SG1 2NY UK; 9 Novel Human Genetics, GSK R&D, Stevenage, SG1 2NY UK

**Keywords:** Adaptive immunity, Gene regulation in immune cells, CD4-positive T cells, Systems analysis

## Abstract

Naïve CD4^+^ T cells coordinate the immune response by acquiring an effector phenotype in response to cytokines. However, the cytokine responses in memory T cells remain largely understudied. Here we use quantitative proteomics, bulk RNA-seq, and single-cell RNA-seq of over 40,000 human naïve and memory CD4^+^ T cells to show that responses to cytokines differ substantially between these cell types. Memory T cells are unable to differentiate into the Th2 phenotype, and acquire a Th17-like phenotype in response to iTreg polarization. Single-cell analyses show that T cells constitute a transcriptional continuum that progresses from naïve to central and effector memory T cells, forming an effectorness gradient accompanied by an increase in the expression of chemokines and cytokines. Finally, we show that T cell activation and cytokine responses are influenced by the effectorness gradient. Our results illustrate the heterogeneity of T cell responses, furthering our understanding of inflammation.

## Abstract (web summary)

Cytokines critically control the differentiation and functions of activated naïve and memory T cells. Here the authors show, using multi-omics and single-cell analyses, that naïve and memory T cells exhibit distinct cytokine responses, in which an ‘effectorness gradient’ is depicted by a transcriptional continuum, which shapes the downstream genetic programs.

## Introduction

The communication between immune cells is mediated by cytokines, which promote the differentiation of cells into effector cell types^1,2^. In particular, upon activation, naïve CD4^+^ T cells are polarized by cytokines into T helper (Th) phenotypes, including Th1, Th2 and Th17. These secrete IFN-γ, IL-4, and IL-17, respectively^3–6^. T helper cells in turn coordinate the downstream response of other immune cells (e.g. CD8^+^ T cells, macrophages and B cells)^7^. Previous studies have increased our understanding of cytokine-induced polarization^8–17^. Nonetheless, most studies focus exclusively on naïve CD4^+^ T cells. This is due to the premise that, once polarized, the phenotype acquired by CD4^+^ T cells remains stable. Recent studies have challenged this idea, showing that cytokines can reprogram previously polarized cells^2,18,19^. For example, IL-6 converts regulatory T (Treg) cells to a pathogenic Th17-like phenotype under arthritic conditions^20^. Furthermore, Th17 cells upregulate *TBX21* and IFN-γ in response to Th1-polarizing cytokines^21^, and infection-induced Th17 cells can secrete Th1 cytokines^22^. These observations highlight the plasticity of CD4^+^ T cells and suggest that memory cells respond to cytokines. Furthermore, genetic studies have implicated memory T cells in many complex immune diseases^23–25^, making it crucial to understand their response to cytokines. However, studying the effects of cytokines on memory T cells is challenging because memory cells comprise multiple subpopulations^26–28^.

Here, we characterized the response of naïve and memory CD4 T cells to five different cytokine combinations at two different time points following stimulation, profiling bulk and single-cell gene expression. At the single-cell level, we show that CD4^+^ T cells form a transcriptional continuum which progresses from the naive to the central and effector memory phenotypes. This progression is accompanied by increased expression of effector molecules and influences the response to activation and cytokine-polarization. Our results provide a new framework for studying naive and memory T cell activation.

## Results

### Study design

**Fig. 1:**
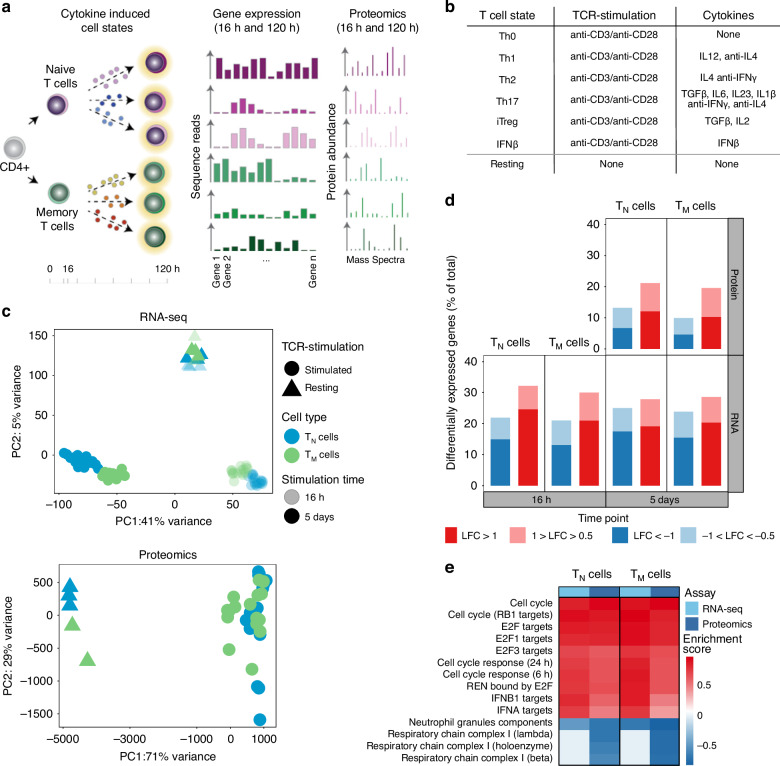
TCR/CD28-activation induces cell type specific gene expression programs in CD4^+^ T cells. **a** Overview of the experimental design. **b** List of cytokine conditions. **c** PCA plots from the whole transcriptome (upper panel) and proteome (lower panel) of T_N_ and T_M_ cells. Different colors correspond to cell types and different shades to stimulation time points. PCA plots were derived using 47 naive and 47 memory T cell samples for RNAseq and 21 naive and 19 memory T cell samples for proteomics. **d** Gene expression changes at the RNA and protein levels by comparing TCR/CD28-activated (Th0) cells to resting cells. Up-regulated genes are in red and down-regulated genes are in blue. Different shades indicate different fold-change thresholds. **e** A selection of significantly enriched pathways (with enrichment scores > 0.7) from genes and proteins differentially expressed after 5 days of activation using the 1D enrichment method. Source data are provided as a Source Data file.

To investigate the effects of cytokines on human naive (T_N_) and memory (T_M_) CD4^+^ T cells (Supplementary Fig. 1A), we stimulated cells with anti-CD3/anti-CD28 coated beads in the presence of different cytokine cocktails (Fig. 1a, b and Supplementary Data 1). We polarized T_N_ and T_M_ toward four T helper phenotypes (Th1, Th2, Th17, and iTreg), as well as including IFN-β due to its role in multiple sclerosis^29,30^. To distinguish T cell responses to TCR/CD28-activation from responses induced by cytokines, we stimulated cells with anti-CD3/anti-CD28 beads in the absence of cytokines (Th0). Finally, we cultured cells in the absence of stimulation or cytokines (resting cells). We profiled gene expression (RNA-seq) 16 h (before cell proliferation) and 5 days after stimulation (when cells have acquired an effector phenotype). To comprehensively characterise cellular states at the late time point, we also profiled the whole proteome (liquid chromatography-tandem mass spectrometry, LC-MS/MS), and single-cell transcriptomes (scRNA-seq) (Methods).

### Activation induces cell type specific responses in T_N_ and T_M_

To understand T_N_ and T_M_ responses to T cell activation (TCR/CD28-activation), we compared the transcriptomes of activated and resting cells. The main source of variation across the transcriptome and proteome was T cell activation, with resting cells separating from activated cells (Fig. 1c). Activated cells clustered by duration of stimulation (16 h and 5 days) and cell type (T_N_ and T_M_), suggesting that the response to T cell activation is dynamic and cell type specific (Fig. 1c). We then assessed differential gene expression between resting and activated (Th0-stimulated) T_N_ and T_M_ (Fig. 1d, Supplementary Data 2 and 3). At the RNA level, 8333 and 7181 genes (40% of genes) were differentially expressed after 16 h in T_N_ and T_M_, respectively. This number was comparable after five days (7705 and 7544 in T_N_ and T_M_). At the protein level, 4009 and 3443 proteins were differentially expressed (35% of proteins) after five days in T_N_ and T_M_, respectively. These genes were enriched in cell cycle progression and type I IFN response (Fig. 1e and Supplementary Data 4). Conversely, T_N_ and T_M_ downregulated components of the respiratory chain complex (Fig. 1e), in line with previous observations that T cell activation induces proliferation and metabolic changes to support effector responses^31^. These observations were consistent between RNA and protein.

### Cytokines induce cell type specific responses in T_N_ and T_M_

**Fig. 2:**
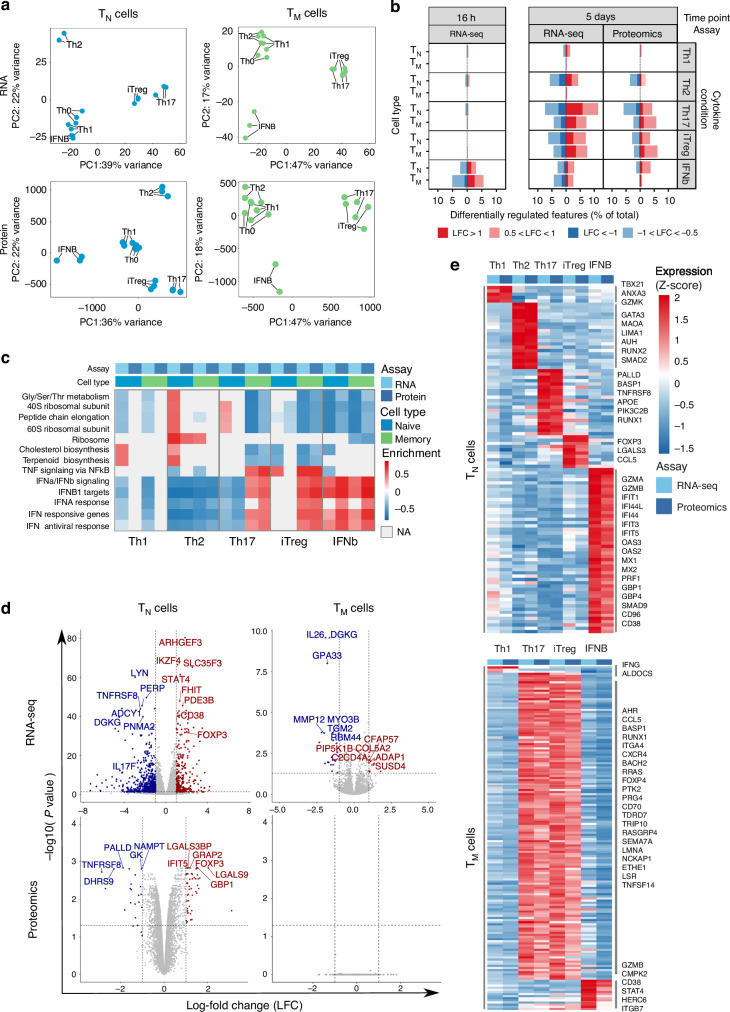
Cytokines induce cell type specific gene expression programs in CD4^+^ T cells. **a** PCA plot from the full transcriptome and proteome of T_N_ and T_M_ cells following five days of cytokine stimulations. Only stimulated cells were included in this analysis. PCA plots were derived using 20 naive and 21 memory T cells samples RNAseq and 18 naive and 17 memory T cells for proteomics. **b** Gene expression changes at the RNA and protein levels from pairwise comparisons between cytokine-stimulated cells and Th0-stimulated cells. Up-regulated genes are in red and down-regulated genes are in blue. Different shades indicate different fold-change thresholds. **c** Selection of significantly enriched pathways (with enrichment scores > 0.7 in at least one cytokine condition) estimated using differentially expressed genes and proteins with the 1D enrichment method. Colors correspond to the enrichment score estimated for each pathway. For the pathways and conditions indicated in light gray, not enough genes were detected to reliably estimate enrichment scores (NAs). **d** Volcano plots highlighting significant differences in gene and protein expression between Th17 and iTreg-stimulated T_N_ and T_M_ cells. Red indicates expression upregulation in iTreg with respect to Th17-stimulation, blue indicates expression upregulation in Th17 with respect to iTreg-stimulation. Labels were added to *IL17*, *FOXP3* and the top 20 most differentially expressed genes. **e** Cell state-specific gene signatures defined using jointly RNA and protein expression. Colours encode normalized (Z-scored) gene and protein expression levels. Example genes for each signature are labelled. Source data are provided as a Source Data file.

We next investigated how cytokines modulate gene expression in T_N_ and T_M_. We performed PCA on the proteome and transcriptome, treating time points and cell types independently. While there were few cytokine effects at 16 h (Supplementary Fig. 1B), we observed clear clustering by cytokine condition at five days (Fig. 2a) in both transcriptome and proteome. We next compared stimulated cells exposed to cytokines to Th0-stimulated cells (Supplementary Data 2 and 3). Most cytokine-induced changes were only apparent after five days of stimulation (Fig. 2b), with the exception of IFN-β. For example, Th17-stimulation induced 42 differentially expressed genes after 16 h in TN, compared to 1818 differential genes induced after 5 days. In contrast, IFN-β induced a large number of early transcriptional changes (357 genes at 16 h and 329 after 5 days in T_N_), reflecting its role in the fast response to viruses. These results suggest that early changes in gene expression are dominated by T cell activation, while the expression programs of differentiated Th cells are only apparent at later stages of stimulation. This implies that cytokine polarization occurs after the initiation of T cell activation.

Since cytokine-induced effects manifested five days after stimulation, we focused on this time point to further elucidate changes in gene and protein expression driven by different cytokines. The number of cytokine-induced changes in RNA and protein was comparable between T_N_ and T_M_ (Fig. 2b). However, Th2-stimulation triggered different responses between the two cell types, resulting in differential expression of 944 genes in T_N_ compared to 49 in T_M_. We observed the same trend at the protein level, where 290 proteins were differentially expressed in T_N_ but no differences were detected in T_M_ (Fig. 2b), although T_M_ expressed comparable levels of the IL-4 receptor (Supplementary Fig. 2A). This suggested that T_M_ cannot polarize toward the Th2 phenotype.

We next sought to translate these observations to cellular functions. We observed that the genes and proteins differentially expressed upon cytokine stimulation were enriched in relevant pathways (Fig. 2c and Supplementary Data 4). Stimulation with IFN-β induced upregulation of the type I IFN response in both T_N_ and T_M_, while Th2-polarization of T_N_ suppressed this pathway, likely reflecting that Th2-polarization involves IFN-γ blockade. These effects were concordant between RNA and proteins (Supplementary Fig. 3). Furthermore, Th1-stimulation of T_N_ induced metabolic changes such as increased cholesterol and terpenoid synthesis, while Th2-stimulation increased the expression of genes involved in amino acid metabolism (Fig. 2c). Interestingly, some pathways showed opposite effects between T_N_ and T_M_. For example, while Th17-stimulation of T_N_ induced downregulation of the type I IFN response, Th17-stimulation of T_M_ increased the activity of this same pathway. We observed a similar pattern upon iTreg-stimulation, with type I IFN response upregulated in T_M_ but not in T_N_ (Fig. 2c). These observations suggest that Th17 and iTreg-stimulation induce different cell states in T_N_ than in T_M_.

Th17 and iTreg cells have been linked to autoimmune inflammation and immune suppression. Polarization to both of these cell states requires the presence of TGF-β and there is evidence of interconversion between them^20^. Consistent with these observations, we found that Th17 and iTreg-stimulated T_M_ cells were more similar to each other than to other cell states and overlapped in PCA space (Fig. 2a). This similarity was captured by both proteome and transcriptome. In contrast, in T_N_ the two cytokine-induced cell states formed separate groups. Importantly, both cell types expressed comparable levels of the TGF-β and IL2 receptors (Supplementary Fig. 2B, C). To test whether Th17 and iTreg-stimulation induced the same phenotype in T_M_, we compared gene expression between the two cell states (Fig. 2d, Supplementary Data 2 and 3). Only 42 genes and no proteins were differentially expressed between the two cytokine conditions in T_M_ (LFC > 1 at 0.05 FDR for RNA-seq and LFC > 0.5 at 0.1 FDR for proteomics). In contrast, in T_N_ 733 genes and 455 proteins were differentially expressed between iTreg and Th17-stimulated cells (Fig. 2d). In particular, iTreg-stimulated T_N_ expressed higher levels of *FOXP3*, *IKZF4*, and *LGALS3*, while Th17-stimulated T_N_ expressed higher levels of *IL17F*, *TNFRSF8*, and *PALLD*. Therefore, while T_N_ acquire different phenotypes upon Th17 and iTreg-polarization, both cytokine conditions polarize T_M_ toward the same cell state.

### Cell state-specific gene signatures

Our results suggested that cytokines act in a cell type specific manner to induce five cell states in T_N_ (Th1, Th2, Th17, iTreg, and IFN-β) and three in T_M_ (Th1, Th17/iTreg and IFN-β, with no detectable Th2 response). As we observed high correlation between RNA and protein expression (Supplementary Fig. 3A–C) we applied a multi-omics approach which leveraged both layers of information to derive cell state gene signatures (Methods). In brief, we identified differentially expressed RNA-protein pairs and asked if any of these pairs were present at a higher level in one cell state compared to the rest, obtaining cytokine-specific proteogenomic signatures (Methods). The identified signatures were sensitive to relative changes in both RNA and protein, thus increasing our confidence that these are true cytokine-induced effects.

In T_N_ we identified 105 signature genes across the five cell states (five genes for Th1, 20 for Th2, 20 for Th17, 10 for iTreg, and 50 for IFN-β) (Fig. 2e and Supplementary Data 5). The T_N_ IFN-β signature contained known antiviral genes involved in RNAse L induction (*OAS2*, *OAS3*), GTPase activity (*MX1*, *MX2*) and cell lysis (*GZMA*, *GZMB*)^32^. Signatures of other T_N_ states also included known hallmark genes, such as *GATA3* (Th2), *TBX21* (Th1), and *FOXP3* (iTreg) (Fig. 2e), validating our approach. Additionally, we observed signature genes for Th1 (*ANXA3*), Th2 (*MAOA, LIMA1, MRPS26*), Th17 (*TNFRSF8, RUNX1, PALLD*), and iTreg (*LMCD1, LGALS3, CCL5*) which have not been described in the context of cytokine polarization.

We performed the same analysis for T_M_, where we identified 162 signature genes across the three cell states (three for Th1, 145 for Th17/iTreg and 14 for IFN-β genes) (Fig. 2e and Supplementary Data 5). Since Th17 and iTreg-stimulated T_M_ overlapped on both the RNA and protein levels, we treated them as one phenotype. Several Th17/iTreg T_M_ signature genes were present in the iTreg and Th17 signatures derived from T_N_ (*CCL5, LGALS3, TNFRSF8*) or had been previously linked to one of the two phenotypes in the literature (*BACH2, BATF3, AHR*), suggesting that Th17/iTreg-stimulated T_M_ might have overlapping functions with the Th17 and iTreg states in T_N_. These signatures provide a valuable resource for future follow-up studies in specific biological contexts or disease settings.

### Single cell RNA-seq reveals a T cell effectorness gradient

Our results showed that the gene expression programs induced in response to cytokines differ substantially between T_N_ and T_M_. Whilest T_N_ constitute a uniform cell population, T_M_ are composed of subpopulations including central (T_CM_) and effector (T_EM_) memory cells, as well as effector memory cells re-expressing CD45RA (T_EMRA_). Given this heterogeneity, we speculated that the observed differences in cytokine responses could be explained in two ways: (i) T_M_ as a whole are unresponsive to certain cytokines, or (ii) a subset of T_M_ responds to cytokines, but bulk gene expression profiles are dominated by a large proportion of unresponsive cells. To address this, we profiled single-cell gene expression in 43,112 T_N_ and T_M_, which included resting, Th0-, Th2-, Th17-, and iTreg- stimulated cells.

**Fig. 3:**
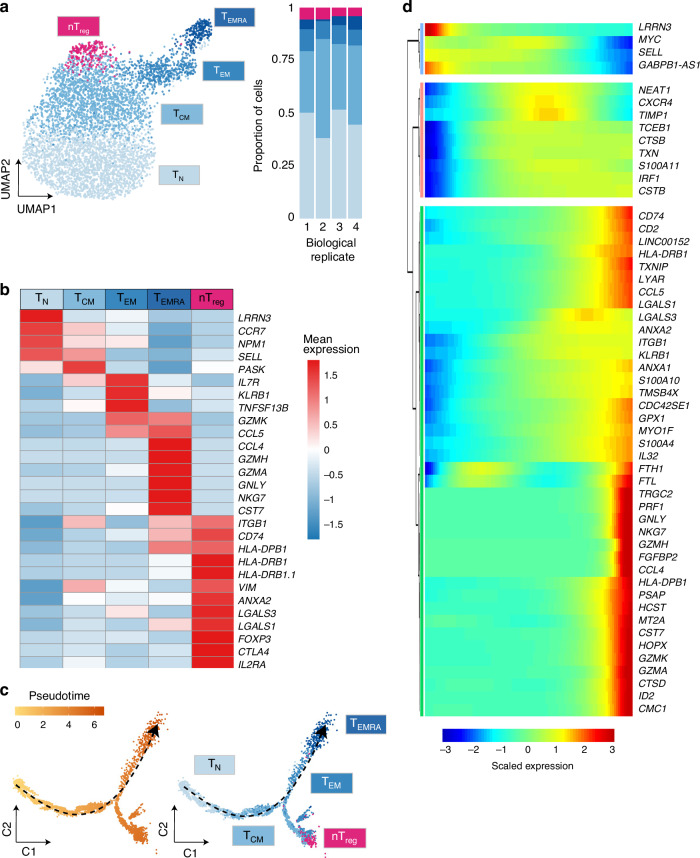
Effectorness gradient in resting CD4^+^ T cells. **a** UMAP of single-cell RNA-seq data from resting T cells. Colors represent cells in the five clusters defined using top variable genes and unsupervised clustering. Bar plots represent the proportion of cells assigned to different clusters in each biological replicate. **b** Gene markers of each cell cluster (Wilcoxon rank sum test) combined with well known markers from the literature. Colors encode the mean expression of each gene in each cluster. **c** Branched pseudotime trajectory, each cell is colored by its pseudotime value (left panel) or its cluster label (right panel), as determined in **a**. **d** Heatmap of genes variable along the pseudotime trajectory (from Monocle). The X axis represents cells ordered by pseudotime (from left to right) and different colors correspond to the scaled (Z-scored) expression of each gene in each cell. Source data are provided as a Source Data file.

First, we isolated T_N_ and T_M_ from four healthy individuals and quantified gene expression in the resting state using droplet-based scRNA-seq^33^, with all replicates for the same cell type in a single reaction (Methods and Supplementary Fig. 4). In total, we profiled 5269 resting T cells (2159 T_N_ and 3110 T_M_ respectively), capturing an average of 1146 genes per cell. We identified 64 highly variable genes, which we used for dimensionality reduction, embedding with the uniform manifold approximation (UMAP)^34^, and unsupervised clustering (Methods). We identified five distinct groups of cells (Fig. 3a) which we annotated as T_N_, T_CM_, T_EM_, T_EMRA_, and natural T regulatory (nTreg) cells based on the expression of well established cell type markers (Fig. 3b and Supplementary Data 6). T_EMRA_ cells showed a distinct transcriptional profile (eg. *PRF1*, *CCL4*, *GZMA*, *GZMH*), consistent with previous observations^27^. The proportions of cells in each cell subpopulation were comparable across individuals (Fig. 3a). We observed that T_N_ were a homogeneous group of cells. However, a small percentage of T_EMRA_ cells were isolated as T_N_ (as they re-express CD45RA) and could only be identified at the single-cell level.

Additionally, our results showed that CD4^+^ T cells do not comprise discrete subpopulations. Instead, CD4^+^ T cells comprise one major population with multiple interrelated transcriptional states. To investigate the relationships between these states we applied pseudotime analysis^35^ and observed that the cells formed a continuous progression starting in T_N_ and gradually progressing towards T_M_ (Fig. 3c). The cells at the beginning of this trajectory expressed high levels of naïve markers (e.g. *SELL, CCR7* and *LRRN3*), while the end of the trajectory was enriched in cells expressing cytotoxic molecules (e.g. *GZMA, GZMB*, and *PRF1*) and cytokines and chemokines (IL32, CCL4, and CCL5) (Fig. 3d and Supplementary Data 7). The cluster labels confirmed that CD4^+^ T cells formed a natural progression that started with naive T cells (T_N_), advanced towards central (T_CM_), then effector (T_EM_) and finally highly effector (T_EMRA_) memory T cells (Fig. 3c), with nTreg cells branching out. Based on these observations, we reasoned that the observed CD4^+^ T cell continuum reflected the potential of cells to initiate a rapid and robust response upon stimulation, i.e. cells which express more chemokine and cytokine in the resting state will be able to rapidly secrete them upon stimulation. We refer to this property as effectorness.

Based on our observations, we reasoned that T cell effectorness could be linked to the activation history of cells and accompanied by increased TCR clonality. We tested this using a public data set containing matched single-cell transcriptomes and paired TCR-sequences of CD4^+^ T cells from peripheral blood of colorectal cancer patients^36^ (Methods). We retrieved all cells corresponding to the subpopulations identified in our data-set (T_N_, T_CM_, T_EM_ T_EMRA_, and nTreg; Supplementary Fig. 5A) and performed pseudotime ordering. This identified the same progression found in our data (Supplementary Fig. 5B), driven by a similar set of genes (Supplementary Fig. 5C). Next, we assigned an effectorness value to each T cell (Methods) and leveraged the paired TCR-sequences to test if effectorness was associated with clonal expansion. Cells with high effectorness values had reduced TCR clonotype diversity and higher numbers of highly expanded clones (Methods and Supplementary Fig. 5D). To assess if this correlated with antigen specificity (i.e. effectorness being driven by a small number of clones specific for the same antigen), we used grouping of lymphocyte interactions by paratope hotspots (GLIPH)^37^, which groups together TCR-sequences predicted to recognize the same peptide-MHC complex (Methods). We observed that even cells in the highest effectorness range (effectorness > 0.7, which corresponds to T_EMRA_) comprised multiple independent TCR clonotypes, each of which was predicted to interact with a different peptide (Supplementary Fig. 5E). Thus, effectorness is a consequence of T cell activation history and is accompanied by clonal expansion, irrespective of antigen-specificity.

### Effectorness shapes the response of T cells to activation

**Fig. 4:**
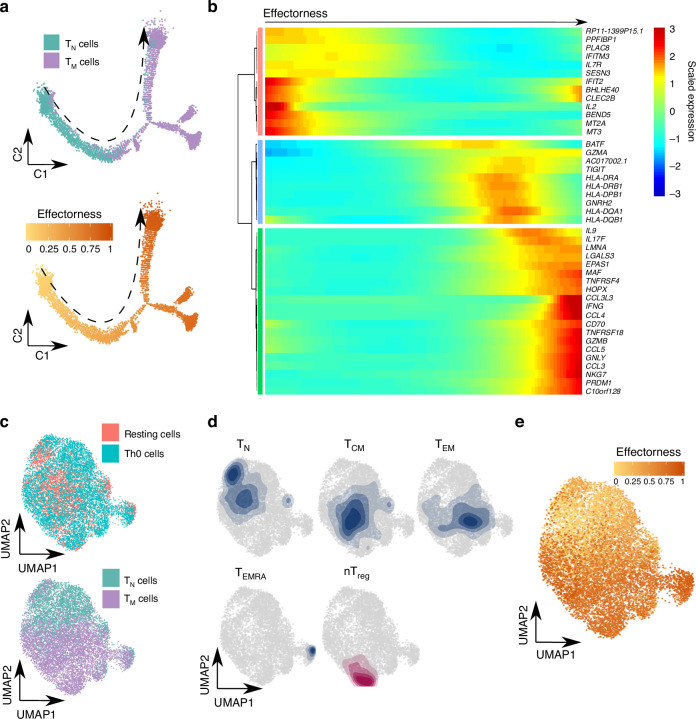
The effectorness gradient is preserved after T cell activation. **a** Th0-stimulated T_N_ and T_M_ cells ordered in a branched pseudotime trajectory. Cells are colored by cell type (top panel) or effectorness (bottom panel). **b** Heatmap of the most variable genes along the Th0 pseudotime trajectory (Methods). The *x* axis represents cells ordered by effectorness (from left to right) and colors correspond to the scaled (Z-scored) expression of each gene in each cell. **c** Mapping of Th0-stimulated cells to their corresponding resting T cell populations. Integration of resting and Th0-stimulated cells using canonical correlation analysis (Methods) followed by UMAP embedding. Cells are colored by activation status (resting vs Th0, top panel) or cell type (bottom panel). **d** Density plots highlighting the location of cell clusters as defined in resting state. **e** UMAP embedding color-coded by the effectorness values of resting and stimulated cells. Source data are provided as a Source Data file.

We next assessed if T cell effectorness influenced responses to TCR/CD28-activation. We quantified single-cell gene expression in Th0-stimulated T_N_ (2543 cells) and T_M_ (4766 cells), with an average of 3677 genes per cell. Pseudotime ordering of Th0-stimulated cells revealed an equivalent trajectory to that observed in resting cells, with T_N_ gradually progressing towards T_M_, accompanied by increased expression of similar genes to those in the resting state (i.e. *CCL3, CCL4, GZMB, GMZA*; Fig. 4a, b). Furthermore, some genes were associated with the trajectory only upon TCR/CD28-activation (i.e. IFNG and IL2).

The expression of classical T_CM_ and T_EM_ markers, such as *SELL* and *CCR7*, significantly changes following activation (Supplementary Fig. 6), making the annotation of T_M_ subpopulations challenging. Therefore, we used the transcriptome of resting T cells to build a reference map and mapped Th0-stimulated cells to the unstimulated reference to annotate subpopulations upon stimulation (Methods). Following integration of resting and stimulated cells (Fig. 4c) we observed that the pattern in the resting state persisted upon activation, with a gradual progression of cells from T_N_ to T_CM_, T_EM_ and T_EMRA_ cells (Fig. 4d and Supplementary Data 7). Finally, we integrated these results with effectorness scores, which were calculated independently for resting and Th0-stimulated cells. Independently of stimulation, cells with the lowest effectorness localized to the T_N_ area, while cells with higher effectorness localized to the T_CM_, T_EM_ and T_EMRA_ areas (Fig. 4e). Thus, we concluded that T cell effectorness is detectable before and after T cell activation. Moreover, we found that T cells of different effectorness respond differently to TCR/CD28-stimulation, differentially regulating cytokines such as *IFNG* and *IL2*.

### Effectorness shapes the response of T cells to cytokines

We next assessed if T cell effectorness influenced cell responses to cytokine polarization. To do so, we analyzed single-cell gene expression in Th2, Th17 and iTreg-stimulated T_N_ and T_M_. We separately ordered Th2, Th17 and iTreg cells into branched pseudotime trajectories (Supplementary Fig. 7) and showed that cells exposed to cytokine polarization also preserve their effectorness properties. (Supplementary Fig. 7 and Supplementary Data 7).

**Fig. 5:**
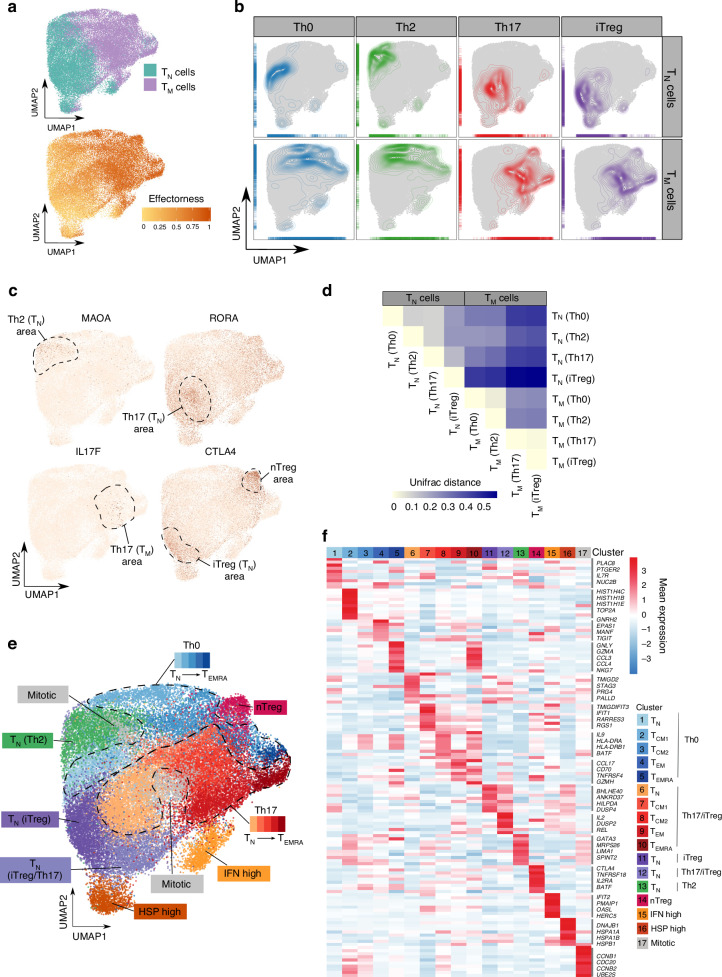
The effectorness gradient persists after cytokine-induced T cell polarization. **a** UMAP embedding of stimulated T cells into a two-dimensional space. Cells are colored by cell type (top panel) or effectorness value (bottom panel). **b** Density plots highlighting cells based on the cytokine stimulations. **c** Expression of selected cytokine-specific markers obtained from our bulk RNA-seq analysis. Cells are colored by their expression level of each marker gene. **d** UniFrac distances between T_N_ and T_M_ cells exposed to different cytokines summarized in a correlation plot. **e** Annotation of 17 cell clusters identified from unsupervised clustering using the top variable genes. Each cluster is annotated based on either the genes with highest expression or the effectorness and cytokine condition of the cells contained in it. **b** Heatmap of the top 10 markers of each cluster (Wilcoxon rank sum test). Colors encode the mean expression of each gene in each cluster. Labels were added to a number of example genes for each cluster. Source data are provided as a Source Data file.

We then merged the data obtained from Th0 and cytokine conditions in a single dataset. To compare the effectorness values after merging, we derived a unified effectorness measurement by scaling the values inferred from independent cytokine-specific trajectories (Methods). The final data set contained transcriptomes of 37,843 cells, of which 18,786 were T_N_ and 19,057 were T_M_. T_N_ and T_M_ formed a single group of cells in UMAP space, but separated by cell type into two different areas (Fig. 5a). As expected, this separation correlated with the effectorness gradient (Fig. 5a), suggesting that T cell effectorness is amongst the strongest drivers of gene expression variation.

In addition to their separation by effectorness, T_N_ and T_M_ exposed to different cytokines also localized to different areas of the UMAP space (Fig. 5b), suggesting that cytokine polarization generates distinct T cell states and is a second major driver of transcriptional variation (Fig. 5c). For example, iTreg-stimulated T_N_ cells localized to an area with high expression of *CTLA4*, while the area associated with Th17-stimulated T_N_ showed high *RORA* expression. Similarly, the area enriched in Th17-stimulated T_M_ showed higher levels of *IL17F* (Fig. 5c). Cells also showed higher expression of the corresponding signature genes (Supplementary Fig. 8).

We next asked whether the absence of response to Th2-stimulation in T_M_ was characteristic of the entire population of cells or if a small group of cells responded to Th2-stimulation but was masked by a majority of unresponsive cells. Interestingly, Th0 and Th2-stimulated T_M_ predominantly localized to the same UMAP areas (Fig. 5b). We used UniFrac distances^38^, to formally test if Th0 and Th2-stimulated T_M_ overlapped or formed different groups. A UniFrac distance of 0 indicates that cells from the two groups have exactly the same composition, while a distance of 1 indicates that the groups form entirely separate clusters. We confirmed that Th0 and Th2-stimulated T_M_ overlapped substantially (UniFrac distance = 0.047) (Fig. 5d) indicating that none of the subpopulations of T_M_ were capable of responding to Th2-stimulation. Instead, the observed lack of response was a uniform characteristic of all T_M_.

Our observations from the bulk data also support that T_M_ polarize to the same cell state in response to Th17 and iTreg-stimulation. We confirmed this at the single-cell level, where cells from these two conditions localized to the same UMAP areas. The UniFrac distance between these cell states was 0.015 in T_M_, compared to 0.164 in T_N_ (Fig. 5d). Thus, we concluded that in response to Th17 and iTreg-stimulation T_M_ converge on the same cell state. This is not driven by any subpopulation of T_M_ and is rather a general characteristic of memory T cell biology. Interestingly this population expressed high levels of *IL17F*, suggesting that iTreg-stimulation in T_M_ induces a Th17-like phenotype.

These results suggest that the transcriptome of a single T cell is shaped by the combination of two factors: T cell effectorness and cytokine-stimulation. Despite being separate biological variables, we hypothesized that these two axes of variation could interact to determine the transcriptional profile of each cell. Thus, we performed unsupervised clustering and annotated the resulting clusters based on the effectorness values as well as the cytokines they were exposed to. This resulted in 17 clusters. (Fig. 5e and Supplementary Data 6). For instance, we identified clusters of Th0, Th2, Th17 and iTreg-stimulated T_N_, as well as a cluster formed of similar numbers of Th17 and iTreg-stimulated T_N_, characterised by high expression of TNF-signaling molecules (eg. *IL2, DUSP2, REL, TNF*) (Fig. 5e, f). Moreover, we identified four clusters of Th0-stimulated T_M_, which we annotated as stimulated T_CM_1, T_CM_2 T_EM_ and T_EMRA_ cells. The same was true for Th17/iTreg-stimulated T_M_, which localized into four groups with different effectorness (T_CM_1, T_CM_2, T_EM_, and T_EMRA_). We also identified a group of nTreg cells, which expressed canonical markers, such as *FOXP3, CTLA4*, and *TNFRSF8*. This cluster contained a comparable number of cells from all cytokine conditions, suggesting that these cytokines do not affect the phenotype of nTregs. Finally, we observed a small cluster formed of T_N_ and T_M_ expressing high levels of IFN-induced genes, as well as a cluster expressing heat shock proteins (HSPs) and other markers of cellular stress (Fig. 5e, f). In conclusion, T cell effectorness and cytokine-induced polarization act jointly to modify the transcriptome of single T cells.

**Fig. 6:**
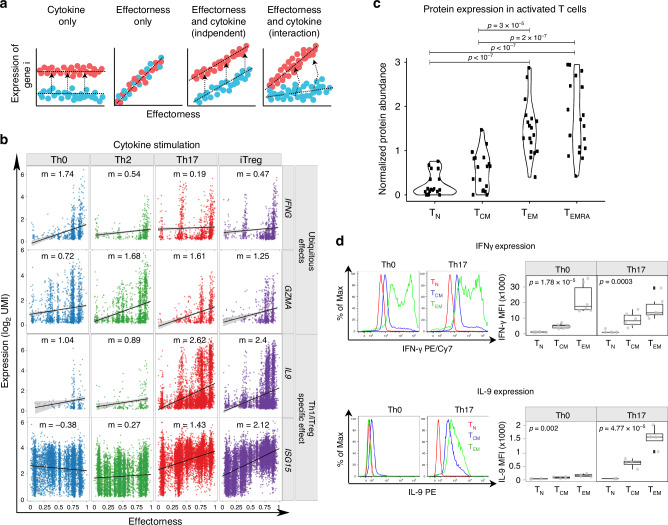
Interactions between effectorness and cytokine condition regulate gene expression. **a** Schematic representation of the gene expression interaction model. Effectorness and cytokine conditions were incorporated into a linear model with an interaction term (Methods). Genes were assigned to four groups: genes induced by cytokine-stimulation regardless of effectorness (left panel), genes which correlate with effectorness regardless of cytokine-stimulation (central left panel), and genes which correlate with both effectorness and cytokine-stimulation either independently (central right panel) or through an interaction (right panel). **b** Plots of gene expression (*y* axis) as a function of effectorness (*x* axis), with cells stratified by cytokine condition. Two example genes significantly associated with effectorness regardless of cytokine conditions (top panels) and two example genes with a strong interaction between effectorness and Th17 or iTreg-stimulation (bottom panels). Each dot represents a single-cell. **c** Protein expression levels for 18 genes associated with effectorness in activated T_N_, T_CM_, and T_EM_ and T_EMRA_ cells regardless of cytokines condition (genes used in the analysis: *ACTB, CCL3, CCL5, CTSW, GNLY, GZMA, GZMB, HLA-DPB1, HLA-DQA1, HLA-DRA, HLA-DRB1, HOPX, IFNG, LGMN, LMNA, NFKBIA, TMEM173, TNFRSF18*)). Expression values were obtained from a publicly available quantitative proteomics data set (Methods). Significance was calculated using one-way ANOVA (*p* = 3.42 × 10^−12^) and group means were compared using Tukey’s Honest Significant Difference test. Only significant *p*-values are shown (*p* < 0.05). Each dot represents an individual gene (*n* = 18). **d** Levels of IFNγ and IL-9 protein in Th0 and Th17-stimulated T_N_, T_CM_, and T_EM_ cells as assessed by flow cytometry. Representative cytometry histograms of IFNγ and IL-9 expression (left panels) and mean fluorescence intensity (MFI) of cytokine-expressing cells (right panels). *P*-values were calculated using one-way ANOVA. Each dot represents a biologically independent sample. Number of samples: 6 (IFNγ) and 4 (IL-9). Boxplot centers represent median values, while the boxplot bounds represent the 25% and 75% quantiles. Boxplot whiskers represent the 25% quantile − 1.5 × interquartile range (IQR) and the 75% quantile + 1.5 × IQR, respectively. Source data are provided as a Source Data file.

To understand how effectroness shapes T cell response to cytokines, we modelled gene expression as a function of effectorness, cytokine condition (i.e. Th0, Th2, Th17 or iTreg) and the interaction between them (Methods). Our model accounted for four possible mechanisms (Fig. 6a): (i) gene expression modulation by a cytokine irrespective of effectorness, (ii) gene expression modulation as a function of effectorness irrespective of the cytokine, and gene expression modulation by effectorness and cytokine-stimulation acting (iii) independently or (iv) jointly (interaction effect). We identified 210 genes significantly associated with effectorness (estimated FDR-corrected *p* value for *β* < 0.05, see Methods and Supplementary Data 8). Of these, the majority (203 genes) were further regulated by cytokines. In particular, 12 genes showed independent effects of cytokine-stimulation and effectorness (estimated FDR-corrected *p* value for *γ* < 0.05, see Methods and Supplementary Data 8), while 191 showed an interaction effect (estimated FDR-corrected *p* value for *δ* < 0.05, see Methods and Supplementary Data 8). Within the genes with interaction effects, 12 showed an effectorness dependency only in the presence of a given cytokine, while 179 showed effectorness dependency ubiquitously (across all cytokine conditions, Supplementary Data 8), with the strength of this effect regulated by cytokines.

We next filtered genes by their effect sizes and identified 24 genes with a strong effectorness dependency across all cytokine conditions (**|β**| > 0.5, see Methods). These included *TNFRSF4* (encoding for OX40), which is known to be critical in the maintenance of memory T cell responses^39^, as well as effector molecules such as granulysin (*GNLY*), *GMZA*, *CCL3* and *IFNG* (Fig. 6b and Supplementary Data 8). The expression of these genes increased proportionally to T cell effectorness. In addition, we identified 37 and 16 genes strongly associated with effectorness and with particularly large effects upon Th17 and iTreg-stimulation, respectively (|**δ**| > 0.5 for the respective cytokine, see Methods). These genes included cytokines like *IL2* (which decreased with effectorness upon Th17 and iTreg-stimulation) and *IL9* (which increased with effectorness in these conditions) (Fig. 6b and Supplementary Data 8). Moreover, genes induced by type I IFNs (eg. *ISG15, IFIT1, IFIT2, IFIT3*) also increased with effectorness upon iTreg and Th17-stimulation. This is in line with our observations from bulk RNA and protein expression, where we found that the type I IFN response was differentially regulated in T_N_ and T_M_ in response to Th17 and iTreg-stimulation (Fig. 2c).

To validate these results, we identified all the genes which contribute to the effectorness gradient and which have a large effect across all cytokine conditions (Supplementary Data 8). We then assessed their expression levels in a proteomics study which profiled resting and activated immune cell populations^40^. Of the 24 effectorness-associated genes with large and ubiquitous effects, 18 were present in this data set and followed the same trend observed in our scRNA-seq data (Fig. 6c). We also experimentally validated two cytokines (IFNγ and IL-9), which showed strong association with effectorness either across all conditions or upon Th17/iTreg stimulation. We isolated CD4^+^ T_N_, T_CM_ and T_EM_ from blood (Supplementary Fig. 9), performed Th0 and Th17-stimulation and quantified the production of IFNγ and IL-9 upon restimulation (Methods). As we observed in scRNAseq, the levels of IFNγ increased proportionally to effectorness in both Th0 and Th17-stimulated cells (Fig. 6d), while the levels of IL9 only marginally correlated with effectorness in Th0 cells, but substantially increased with effectorness in Th17-stimulated cells (Fig. 6d). This confirmed our observations from the transcriptome and suggested that key T cell functions such as cytokine secretion are under the control of both effectorness and environmental cues, and that these two factors can interact.

In summary, we showed that CD4^+^ T_N_, T_CM_, T_EM_, and T_EMRA_ cells form a transcriptional continuum characterized by an effectorness gradient. This gradient is determined by the expression of cytokine, chemokine, and granzyme genes and affects the response of cells to cytokine polarization.

## Discussion

Cytokines have been extensively studied in the context of naïve T cells, but the response of memory T cells to cytokines remains understudied. Here we analyzed the effects of cytokines on T_M_ gene expression and compared these to the responses of T_N_. We demonstrate that early gene expression changes in both T_N_ and T_M_ cells are dominated by the response to TCR and CD28, while cytokine-induced changes are apparent only at later stages of stimulation. This suggests that polarization to T helper subsets occurs after the initiation of T cell activation and fine-tunes the response of T cells.

Our multi-omic data further show that the response to cytokines differs between T_N_ and T_M_. While T_N_ respond to all tested cytokine conditions, acquiring a distinctive phenotype, T_M_ do not respond to Th2 polarization. Furthermore, while T_N_ induce hallmark markers such as *FOXP3* and CTLA4 upon iTreg-stimulation, T_M_ converge on the same cell state in response to both Th17 and iTreg-stimulation. This state is characterized by high levels of *IL17F*, suggesting that T_M_ do not acquire a regulatory phenotype upon iTreg polarization. This is particularly relevant in the context of disease, given that T_M_ increase with age^41^, potentially leading to a pro-inflammatory response to TGF-β.

In contrast, the response to IFN-β was conserved between T_N_ and T_M_ and was apparent within 16 h of stimulation. This is in line with type I interferon responses, which are rapidly triggered to prevent viral replication. Both T_N_ and T_M_ upregulated genes involved in RNAse L induction (*OAS2*, *OAS3*)^42^, GTPase activity (*MX1*, *MX2*)^43^, as well as cell lysis (*GZMA*, *GZMB*)^32^.

Using single-cell transcriptomics we show that CD4^+^ T cells form a continuum, characterized by a progression from T_N_ to T_CM_, T_EM_ and T_EMRA_ cells, with nTregs branching out separately. This progression is accompanied by upregulation of chemokine and cytokine genes, suggesting that T cells at the end of this trajectory are poised to initiate a rapid effector response upon activation. We thus refer to this property as effectorness. A similar gradient is present in innate T cells, as shown by a recent study where higher expression of cytokines, chemokines and granzymes negatively correlated with ribosome synthesis and proliferative capacity^44^. Additionally, we show that high T cell effectorness is accompanied by clonal expansion and therefore reflects the activation history of each single-cell. These results agree with observations of CD8^+^ T cells upon viral infection, where exposure to CMV results in T cells that express high levels of CCL4 and perforin^45^. The similarity of this profile with the most effector cells in our study suggests that an equivalent gradient exists in CD8^+^ cells, where chronic exposure to CMV drives repeated clonal expansion, generating highly effector memory cells. Moreover, previous studies suggest this process could be impaired in HIV^45,46^.

The effectorness gradient described here recapitulates observations from tissue-resident immune cells. A previous study described the generation of memory T cells in the fetal intestine, with an equivalent trajectory from T_N_ to T_M_^47^, accompanied by downregulation of *CCR7* and upregulation of cytokines like *IL32*. Thus, our results could explain how T_M_ in tissues could adapt to inflammation.

Finally, we demonstrate that effectorness determines how CD4^+^ T cells respond to TCR/CD28 activation and cytokines, and identify genes that increase proportionally to effectorness upon T cell activation or cytokine polarization. For example, *IFNG* and *IL9* show strong effectorness-dependency in response to TCR/CD28 and Th17/iTreg-stimulation, respectively. T_M_ are known to upregulate IL-9 in response to TGF-β^48^, a cytokine which reprograms Th2 cells to an IL-9 secreting phenotype^19^. We refine this observation, showing that IL-9 upregulation is driven by T_M_ of higher effectorness (i.e. T_EM_ and T_EMRA_). This is important given the role of TGF-β in Th17 cell biology, and the substantial Th17 diversity in vivo^22^. Our study suggests that cells with high effectorness which infiltrate tissues might respond strongly to the local cytokine environment. Future scRNA-seq studies of inflamed tissues will provide an opportunity to investigate these effects in greater detail directly. Understanding this will be key in the development of drug targets for autoimmune disease, as Th17-cytokines are known to promote inflammation in multiple sclerosis^49–51^.

## Methods

### Cell isolation and in vitro stimulation

Blood samples were obtained from six individuals for the bulk assays (naïve and memory T cells were isolated from three independent individuals, respectively) and from four additional individuals for single-cell RNA-seq. All individuals were healthy males of 56.4 years of age on average (sd = 12.41 years). Human biological samples were sourced ethically and their research use was in accord with the terms of the informed consents under an IRB/EC approved protocol (15/NW/0282). Peripheral blood mononuclear cells (PBMCs) were isolated using Ficoll-Paque PLUS (GE healthcare, Buckingham, UK) density gradient centrifugation. Naïve (CD25− CD45RA + CD45RO−) and memory (CD25− CD45RA− CD45RO+) CD4^+^ T cells were isolated from PBMCs using EasySep® naïve CD4^+^ T cell isolation kit and memory CD4^+^ T cell enrichment kit (StemCell Technologies, Meylan, France) according to the manufacturer’s instructions. T cells were then stimulated with anti-CD3/anti-CD28 human T-Activator Dynabeads® (Invitrogen) at a 1:2 ratio of beads to T cells. Cytokines were added at the same time as the stimulus (see Supplementary Data 1 for a full list of the cytokines used with product details and exact concentrations). Cells were harvested after 16 h and 5 days of stimulation.

### Bulk RNA-sequencing

A total of 3 × 10^5^ cells were resuspended in 500 μl of TRIzol™ and stored at −80 °C until further processing. After samples were thawed at 37 °C, 100 μl chloroform were added and samples were centrifuged for 15 min at 4 °C and 10,000 g. The aqueous phase was collected and mixed at a 1:1 ratio with 70% ethanol (Qiagen). RNA was isolated from this mixture using the RNeasy MinElute Kit (Qiagen), and RNA quality was assessed using a Bioanalyzer RNA 6000 Nano Chip (Agilent Technologies). All samples had an RNA integrity number (RIN) higher than 7, with a mean RIN of 9.35. Finally, sequencing libraries were prepared using the Illumina TruSeq protocol and sequenced on an Illumina HiSeq 2500 platform using V4 chemistry and standard 75 bp paired-end reads.

### Proteomics

Pellets formed of up to 3 × 10^6^ cells were isolated and washed twice with PBS, dried and stored at −20 °C until protein extraction. Cell pellets were then lysed in 150 μl 0.1 M triethylammonium bicarbonate (TEAB) buffer (Sigma Aldrich) supplemented with 0.1% SDS and Halt protease and phosphatase inhibitor cocktail (100X, Thermo #78442). Pulse probe sonication (40% power, 4 °C and 20 s) was performed twice using EpiShear™, after which the samples were incubated for 10 min at 96 °C. Protein from cell lysates was quantified using the quick start Bradford protein assay (Bio-Rad) as specified by the manufacturer’s instructions. Protein samples were finally divided into aliquots of up to 100 μg. Protein aliquots were reduced with 5 mM tris-2-carboxymethyl phosphine (TCEP) buffer (Sigma Aldrich) and incubated for 1 h at 60 °C to reduce disulfide bonds. Iodoacetamide (IAA) was added to a final concentration of 10 mM and samples were incubated for 30 min at room temperature in the dark. Pierce Trypsin (Thermo Scientific) was then added at a mass ratio of 1:30, and samples were incubated overnight for peptide digestion. Digested protein samples were diluted to a total volume of 100 μl in 0.1 M TEAB buffer. TMT reagents (Thermo Scientific) supplemented with 41 μl anhydrous acetonitrile were added to the corresponding peptide samples. After 1 h, the reaction was quenched using 8 μl 5% hydroxylamine. Samples were then combined into a single tube and dried using a speedvac concentrator. Dry samples were stored at −20 °C until fractionation. High pH Reverse Phase (RP) peptide fractionation was performed with the Waters XBridge C18 column (2.1 × 150 mm, 3.5 μm) on a Dionex™ UltiMate 3000 HPLC system. A 0.1% solution of ammonium hydroxide was used as mobile phase A, while mobile phase B was composed of acetonitrile with 0.1% ammonium hydroxide. The TMT-labelled samples were reconstituted in 100 μl mobile phase A, centrifuged and injected into the column, which operated at 0.2 ml/min. The fractions collected from the column were dried with the SpeedVac concentrator and stored at −20 °C until the MS analysis.

Liquid Chromatography-Mass Spectrometry (LC-MS) was performed using a Dionex™ UltiMate 3000 HPLC system (Thermo Scientific) coupled with the Orbitrap Fusion Tribrid Mass Spectrometer (Thermo Scientific). Dried samples were reconstituted in 40 μl 0.1% formic acid, of which 7 μl were loaded to the Acclaim PepMap 100 trapping column (100 μm × 2 cm, C18, 5 μm, 100Ӓ) at a flow rate of 10 μl/min. Multi-step gradient elution was performed at 45 °C using the Dionex™ Acclaim PepMap RSLC capillary column (75 μm × 50 cm, 2 μm, 100Ӓ). A 0.1% solution of formic acid was used as mobile phase A, and a 80% acetonitrile, 0.1% formic acid solution as mobile phase B. Precursors were selected with mass resolution of 120k, AGC 4 × 10^5^ and IT 50 ms were isolated for CID fragmentation with quadrupole isolation width of 0.7 Th. Collision energy was set at 35%. Furthermore, MS3 quantification spectra were acquired with 50k resolution via further fragmentation for the top 7 most abundant CID fragments in the Synchronous Precursor Selection (SPS) mode. Targeted precursor ions were dynamically excluded for 45 s.

Raw data were processed in Proteome Discoverer (v2.2) with SequestHT search engine (Thermo Scientific) using reviewed UniProt^52^ human protein entries for protein identification and quantification. The precursor mass tolerance was set at 20 ppm and the fragment ion mass tolerance was 0.5 Da. Spectra were searched for fully tryptic peptides with maximum 2 miss-cleavages. TMT6plex at N-terminus/K and Carbamidomethyl at C were used as static modifications. Dynamic modifications included oxidation of M and deamidation of N/Q. Peptide confidence was estimated with the Percolator node. Peptide FDR was set at 0.01 and validation was based on q-value and decoy database search. The reporter ion quantifier node included a TMT10plex quantification method with an integration window tolerance of 15 ppm and integration method based on the most confident centroid peak at the MS3 level. Only unique peptides for the protein groups were used for quantification. Peptides with average reporter signal-to-noise <3 were excluded from protein quantification.

### Single-cell RNA-sequencing

Cells were resuspended in RPMI media to obtain a single-cell suspension with high cell viability. Next, cells were stained with a live/death dye (DAPI) and dead cells were removed using fluorescence-activated cell sorting (FACS). Live cells were resuspended in PBS buffer and recounted using AOPI staining and the Nexcelom Cellometer Auto 2000 Cell Viability Counter. Finally, cells from four independent biological replicates were pooled in equal cell numbers into a single-cell suspension for each condition. Cell suspensions were processed for single-cell RNA-sequencing using the 10×-Genomics 3′ v2 kit^33^, as specified by the manufacturer’s instructions. Namely, 1 × 10^4^ cells from each condition were loaded in separate inlets of a 10×-Genomics Chromium controller in order to create GEM emulsions. The targeted recovery was 3000 cells per condition. Emulsions were used to perform reverse transcription, cDNA amplification and RNA-sequencing library preparation. Libraries were sequenced on the Illumina HiSeq 4000 platform, using 75 bp paired-end reads and loading one sample per sequencing lane.

### Flow cytometry

Cells were washed with FACS buffer (PBS buffer supplemented with 1% FCS and 1 mM EDTA) by centrifugation and stained with the respective antibodies. Reactions were incubated for 30 min at 4 °C. Following two washes with FACS buffer, samples were resuspended in 200 μl of FACS buffer and data was acquired using a Fortessa analyser (BD Bioscience). All data were processed with FlowJo (v9.9, TreeStar). Antibodies used in this study: anti-human CD4, APC (BioLegend; Clone: OKT4, Catalog No: 317416, Dilution: 1:100), anti-human CD45RA, Brilliant Violet 785 (BioLegend; Clone: HI100, Catalog No: 304140, Dilution: 1:100), anti-human CD45RO, PE-Cyanine7 (BioLegend; Clone: UCHL1, Catalog No: 304229, Dilution: 1:100), anti-human CD197 (CCR7), (BD Bioscience; Clone: 150503, Catalog No: 561271, Dilution: 1:100), anti-human IFN gamma, PE-Cyanine7 (eBioscience; Clone: 4s.B3, Catalog No: 25-7319-82, Dilution: 1:50), anti-human IL-9, PE (BD bioscience; Clone: MH9A3, Catalog No: 560814, Dilution: 1:50).

### Intracellular cytokine staining

CD4^+^ T cells were obtained from PBMCs using the EasySep human CD4^+^ T cell enrichment kit (StemCell Technologies, Meylan, France). Next, CD4^+^CCR7^+^CD45RA^+^ (T_N_), CD4^+^CCR7^+^CD45RA^−^ (T_CM_) and CD4^+^CCR7^−^CD45RA^−^ (T_EM_) cells were isolated from CD4^+^ T cells via fluorescence activated cell sorting (FACS) using a MoFlo XDP cell sorter (Beckman Coulter) (Supplementary Fig. 9) and polarized to the Th0 and Th17 phenotypes as described above. After five days of stimulation, activated naive and memory T cells were restimulated with 50 ng/ml phorbol 12-myristate 13-acetate (PMA) (Sigma) and 1 μM Ionomycin (Sigma) for five hours in the presence of 10 μg/ml of Brefeldin A (Sigma) at 37 °C. After five hours, cells were fixed and permeabilized using the eBioscience™ Foxp3/Transcription Factor Staining Buffer Set (Thermo Fisher Scientific), according to the manufacturer’s instructions. Cells were resuspended in 50 μl of permeabilization solution and stained for cytokines, and flow cytometry was performed.

### RNA-seq data analysis

Sequencing reads were aligned to the reference human genome using STAR^53^ (v2.5.3) and annotated using the hg38 build of the genome (GRCh38) and Ensembl (v87). Next, the number of reads mapping to each gene was quantified using featureCounts^54^ (v1.22.2). After quantification, reads mapping to the Y chromosome and the major histocompatibility complex (HLA) region (chr6:25,000,000–47,825,000) were removed from the analysis. The final result from this process was a counts table of RNA expression in each sequenced sample.

RNA counts were imported into R (v3.5.1) where normalization for library size and regularized-logarithmic transformation of counts was performed using DESeq2^55^ (v1.19.52). We identified and removed batch effects using limma^56^ (v3.35.15). Exploratory data analysis was performed using ggplot2 (v3.0.0) and the base R functions for principal component analysis. Differential expression analysis was performed with DESeq2. More specifically, pairwise combinations were performed between any two conditions of interest, usually setting either resting or Th0-stimulated cells as controls. Differentially expressed genes were defined as any genes with absolute log-fold changes (LFC) larger than 1 at a false discovery rate (FDR) of 0.05.

### Proteomics data analysis

After quantification, protein abundances were normalised in order to allow comparisons between samples and plexes (mass spectrometry batches). Namely, protein abundance values were normalized to the total abundance of the respective sample (sample-wise normalization) and then scaled to the maximum abundance of the respective protein (protein-wise scaling). Data were then imported into R, where principal component analysis was performed using all the proteins with no missing values (proteins detected in all batches and samples) with base R functions. Finally, differential protein expression was analyzed by performing pairwise comparisons between any two conditions of interest. This was done using the moderated T test implemented in limma’s eBayes function^56^. When testing for differential protein expression, only proteins detected in at least two biological replicates per condition were kept. Multiple testing correction was performed using the Benjamini-Hochberg procedure^57^. Finally, differentially expressed proteins were defined as any proteins with an absolute log-fold change larger than 0.5 at an FDR of 0.1.

### Pathway enrichment analysis

Pathway enrichment analysis was performed using proteomics and RNA-seq data. To do so, genes detected at both the RNA and protein level were identified by matching gene names. Next, genes were ranked by differential gene or protein expression, respectively, compared to either resting or Th0-stimulated T_N_ and T_M_ cells. Finally, pathway enrichment analysis was performed independently in the RNA and protein data using the Perseus software^58^ (v1.6) and the 1D-annotation enrichment method^59^. The enrichment scores indicated whether the RNAs and proteins in a given pathway tended to be systematically up-regulated or down-regulated based on a Wilcoxon-Mann-Whitney test. A term was defined as differentially enriched if it had a Benjamini-Hochberg FDR < 0.05. Firstly, all significantly enriched pathways with absolute enrichment scores higher than 0.25 in both RNA and protein data for the same cell types and cytokine conditions were retrieved. The enrichment scores estimated for these pathways were used to estimate the correlation between RNA and protein using Pearson correlation coefficients (Supplementary Fig. 3C, D). Next, a subset of strongly enriched pathways was selected for visualization in R using the pheatmap package (v1.0.10). This selection included all pathways with an absolute enrichment score higher than 0.7, an FDR < 0.05 which were included in either Reactome, KEGG or CORUM^60–62^ and were of relevance to CD4^+^ T cell biology.

### Cell state specific gene signatures

The correlation between RNA and protein expression was evaluated by estimating log-fold changes (LFCs) with respect to the control (Th0) in each cytokine condition and computing the Pearson correlation between RNA and protein LFCs. This was done both sample-wise and gene-wise. Resting T cells were excluded from this analysis. Furthermore, correlation estimates were also computed at the pathway level based on the pathway enrichment analysis results.

Proteomics and RNA-seq data were used jointly to identify gene signature associated with each cytokine-induced cell state. First, both data sets were matched by gene name to identify a common set of genes detected at both the RNA and protein level. Next, the f-divergence cut-off index (fCI) method^63^ was used to identify genes (RNA-protein pairs) with significant evidence of differential expression given their RNA counts and protein abundances. For any genes detected as significant by fCI, their normalized regularized-log (rlog) RNA counts^55^ and scaled protein abundances were used to calculate specificity scores in RNA and protein datasets, respectively. To do so, replicates from each condition were first averaged. Next, the specificity of each gene in each cytokine-induced cell state was defined by normalizing the expression of each gene or protein to the Euclidean mean across its different cell states, as described elsewhere^23^.

RNA specificity was defined as:

$$S_{i,j,{{\mathrm{RNA}}}} = \frac{{X_{i,j}}}{{\sqrt {\mathop {\sum }_{j = 1}^n X_{i,j}^2} }}$$

Protein specificity was defined as:

$$S_{i,j,{{\mathrm{prot}}}} = \frac{{Y_{i,j}}}{{\sqrt {\mathop {\sum }_{j = 1}^n Y_{i,j}^2} }}$$

Where **X**_**i,j**_ and **Y**_**i,j**_ are the average RNA expression and protein abundance of gene **i** in cytokine condition **j**, respectively, and **n** is the number of cytokine conditions assessed. As the RNA expression and protein abundance are both non-negative values, S_*i,j,*RNA_ and S_*i,j,*prot_ are both ≥0.

Proteogenomic specificity scores were defined as the weighted sum of RNA and protein specificities for each gene:

$$S_{i,j} = W_{{{\mathrm{RNA}}}}S_{i,j,{{\mathrm{RNA}}}} + W_{{{\mathrm{prot}}}}S_{i,j,{{\mathrm{prot}}}}$$

Where **S**_**i,j**_ is the specificity score of gene i in condition j. In order to give the same weight to proteomic and transcriptomic evidence, the RNA and protein weights (**W**_**RNA**_ and **W**_**prot**_) were set to 0.5.

To test which genes were more specific to one cell state than expected by chance, sample labels were randomly permuted and the specificity score was recalculated. Empirical *P* values were computed as the proportion of times the observed specificity score of a gene in a given cell state was larger than the corresponding permuted value. *P* values were corrected for the number of genes tested using the Benjamini-Hochberg procedure^57^. A total of 10,000 permutations were performed. Finally, proteogenomic signatures for each cytokine condition were defined as any genes with a specificity score larger than 0.7 and an FDR-adjusted *P* value lower than 0.1. This analysis was performed separately for naïve and memory T cells. The functions used to derive proteogenomic signatures are publicly available as an R package on GitHub (https://github.com/eddiecg/proteogenomic).

### Single-cell RNA-seq data analysis

Single-cell RNA-sequencing data were processed using the Cell Ranger Single-Cell Software Suite^33^ (v2.2.0, 10×-Genomics). Namely, reads were first assigned to cells and then aligned to the human genome using STAR^53^, using the hg38 build of the human genome (GRCh38). Reads were annotated using Ensembl (v87). Gene expression was then quantified using reads assigned to cells and confidently mapped to the genome.

Because each of the samples consisted of a pool of four individuals, natural genetic variation was used to identify which cells corresponded to which person. A list of common genetic variants was collected, defined as any SNP included in gnomAD^64^ with a minor allele frequency higher than 1% in the Non-Finish European (NFE) population. Next, cellSNP (v0.99)^65^ was used to generate pileups at these SNPs, resulting in one VCF file per sample. This information was then used by Cardelino^65^ (v0.99, now Vireo) to infer which cells belong to the same individual. Any cells which remained unassigned (with <0.9 posterior probability of belonging to any individual) or were flagged as doublets were discarded. In general, over 85% of cells were unambiguously assigned to an individual (Supplementary Fig. 4). This analysis was performed separately for each sample. To identify which individual from a given sample corresponded to an individual in a different sample, results from Cardelino were hierarchically clustered by genotypic distances between individuals. Clustering separated genotypes into four distinct groups, each group corresponding to one of the profiled individuals.

Results from RNA quantification and genotype deconvolution were imported into R and analysed using Seurat (v2.3.4)^66^. Cells with less than 500 genes detected or with more than 7.5% mitochondrial genes were removed from the data set. Cells expressing high levels of hemoglobin genes (i.e. *HBA* and *HBB*) were also removed from the data set, as they likely represent contamination during cell culture or sample processing. Counts were next normalized for library size and log-transformed using Seurat’s default normalization parameters. Next, a publicly available list of cell cycle genes^67^ was used to perform cell cycle scoring and assign cells to their respective stage of the cell cycle. Cell cycle, as well as any known sources of unwanted variation (mitochondrial content, cell size as reflected by UMI content, biological replicate and library preparation batch) were regressed using Seurat’s built-in regression model. Highly variable genes were identified using Seurat and used to perform principal component analysis. The first 30 principal components were used as an input for SNN clustering and for embedding using the uniform manifold approximation and projection (UMAP)^34^. Marker genes for each cluster were identified computationally using the Wilcoxon rank sum test implemented in Seurat. Multiple testing correction was performed using FDR. Cell cycle genes were excluded from this analysis. Moreover, marker genes were required to be expressed by at least 10% of the cells in the cluster at a minimum fold change of 0.25. A total of five clusters were identified in resting cells and 17 clusters were found in stimulated cells. Clusters were manually annotated according to their gene expression pattern, the cytokine which cells in the cluster were exposed to and the presence or absence of hallmark genes compiled from the literature.

UniFrac distance analysis^38^ was used to test if cells exposed to two different cytokine conditions tended to form the same clusters. Pairwise UniFrac distances were computed for all combinations of cytokine conditions using all the cells captured for the respective conditions. The R package scUnifrac (v0.9.6)^68^ was used as it was specifically adapted to deal with scRNA-seq data. All parameters were set to the default values (1000 permutations, nDim = 4, ncluster = 10).

### Pseudotime ordering

Cells were ordered into a branched pseudotime trajectory using Monocle (v2.12.0) and restricting the analysis to the highly variable genes identified by Seurat. This was done separately for each cytokine condition (resting, Th0, Th2, Th17 and iTreg), including both T_N_ and T_M_. This resulted in five condition-specific pseudotime trajectories. Monocle was used to test for a significant correlation between gene expression and pseudotime in each trajectory. A gene was defined as significantly associated with pseudotime if its estimated *q* value was lower than 0.01.

### Mathematical definition of T cell effectorness

The four pseudotime trajectories derived from TCR/CD28 or cytokine-stimulated T cells (Th0, Th2, Th17 and iTreg) were combined into a single numeric variable. To do this, the pseudotime values of cells within each condition were scaled to the range [0, 1] and all cells were combined into a single data set.

### Alignment of resting and Th0-stimulated cells

The single-cell transcriptomes of resting and Th0-stimulated T cells were analyzed separately according to the methods described above. This allowed the identification and annotation of clusters in resting T cells, as well as the estimation of an effectorness value for every cell in both data sets. Next, canonical correlation analysis (CCA)^66^, as implemented in Seurat v2.3.4, was used to identify correlated features between the two conditions and to align resting and Th0-stimulated cells into a common space of lower dimensionality. The first 30 CCA dimensions were used to perform UMAP embedding and visualization of cells. The cluster labels defined for resting T cells, as well as the effectorness values independently estimated for resting and Th0-stimulated cells were examined in these visualizations and used to annotate Th0-stimulated cells based on the corresponding resting T cell annotations.

### Modelling interaction between effectorness and cytokines

The association between gene expression, effectorness and cytokine-stimulation was tested with the lm() function from base R. The expression of each gene was modelled as a linear function of T cell effectorness (a numeric variable in the [0, 1] range) and cytokine-stimulation (a categorical variable with levels Th0, Th2, Th17 and iTreg). An additional term was incorporated which accounted for potential interactions between these two variables, as specified in the following equation:

$$X_{i,j} = \alpha E_j + \beta C_j + \gamma E_j \ast C_j + \varepsilon$$

Where **X** is the expression of gene **i** in cell **j** (log2 of normalized UMIs), **E** the effectorness of cell **j**, **C** the cytokine cocktail cell **j** was exposed to and **ε** a random error term, which was assumed to follow a normal distribution with a mean of zero. The regression coefficients for the intercept, effectorness, cytokine stimulation and the effectorness-cytokine interaction were represented, respectively, by **α**, **β**, **γ** and **δ**. An estimate and a *P* value were derived for each of these coefficients in each tested gene. *P* values were corrected for the number of genes tested using the Benjamini-Hochberg procedure^57^. This analysis was restricted to the top variable genes identified by Seurat. All cells with zero-expression for a given gene were omitted. A coefficient was defined as significant if its corresponding FDR-adjusted *P* value was lower than 0.05.

### Effectorness and TCR repertoire analysis

Pre-processed scRNA-seq data were obtained from a study profiling CD4^+^ and CD8^+^ T cells from colorectal cancer patients^36^. This data set contained matched full-length transcriptomes and paired TCR-sequences for 10,805 single-cells isolated from 12 patients. First, cells annotated by the authors as CD4^+^ T_N_, T_CM_, T_EM_/T_EMRA_ or Treg cells (i.e. CD4_C01-CCR7, CD4_C02-ANXA1, CD4_C03-GNLY and CD4_C10-FOXP3, according to the cluster labels reported in the study) were retrieved. This resulted in 1,513 single-cells which corresponded to the populations detected in our study. Of these cells, 94% were from peripheral blood of patients, the remaining 6% being either T cells from tumors or from adjacent normal tissues. Cells were next processed using the analysis pipelines described above. Namely, highly variable genes were estimated and used for dimensionality reduction, UMAP embedding and unsupervised ordering of cells in branched pseudotime trajectories. Finally, the effectorness value of each single T cell in the data set was defined as its estimated pseudotime value scaled to the [0, 1] range.

TCR clonotype IDs for each single-cell in the study were retrieved. These clonotypes were next used to estimate three diversity metrics: (1) the fraction of unique clones (i.e. the number of unique TCR clonotypes divided by the total number of clonotypes), (2) the Shannon entropy^69^, and (3) the expansion index (defined as 1 - Shannon entropy)^36^ at different ranges of T cell effectorness. To do so, cells were ordered by increasing effectorness (i.e. from the least to the most effector) and the diversity metrics were estimated using a sliding window of size 100 (i.e. first analyzing the cells ranked 1 to 100, next the cells ranked 2 to 101, and so on until reaching the end of the data set). The three diversity metrics, along with the mean effectorness value, were repeatedly calculated within each window. Importantly, the fixed window size allowed us to avoid the need for any normalization by cell numbers. Sliding windows were created using the rollapply() function included in R’s zoo package^70^.

Finally, the amino acid sequence of the TCRβ1 CDR3 region of each cell^71^ was retrieved from the study supplementary data. The grouping of lymphocyte interactions by paratope hotspots (GLIPH)^37^ algorithm was then used to cluster TCR sequences by pMHC specificity based on their CDR3 sequences. The TCR network and clonotype groups inferred by GLIPH were imported into R and visualized using the igraph package^72^, focusing on the clonotypes of cells with an estimated effectorness value > 0.7.

### Analysis of public proteomics data

We downloaded proteomics data from Rieckmann, J. C. et al. (Rieckmann et al.^40^) and calculated mean expression for each protein across donors. Of the 24 effectorness-associated genes with large and ubiquitous effects, 18 were present in Rieckmann et al. data set (ACTB, CCL3, CCL5, CTSW, GNLY, GZMA, GZMB, HLA-DPB1, HLA-DQA1, HLA-DRA, HLA-DRB1, HOPX, IFNG, LGMN, LMNA, NFKBIA, TMEM173, TNFRSF18). The protein abundance was normalized to mean protein abundance across cell types. Significance was calculated using one-way ANOVA and group means were compared using Tukey’s Honest Significant Difference test.

### Reporting summary

Further information on research design is available in the Nature Research Reporting Summary linked to this article.

## Supplementary information

Supplementary Information

Peer Review File

Reporting Summary

Description of Additional Supplementary Files

Supplementary Data 1

Supplementary Data 2

Supplementary Data 3

Supplementary Data 4

Supplementary Data 5

Supplementary Data 6

Supplementary Data 7

Supplementary Data 8

## Source Data

Source Data

## Data Availability

RNA-seq: raw data have been deposited in the European Genome-Phenome Archive (EGA) under the study accession number EGAS00001003823 and the sample accession number EGAD00001005291. scRNA-seq: raw data have been deposited in the European Genome-Phenome Archive (EGA) under the study accession number EGAS00001003215 and the sample accession number EGAD00001005290. Mass spectrometry: raw data have been deposited in the Proteomics Identifications Database (PRIDE) under the accession number PXD015315. Count tables: Files containing RNA-seq counts, scRNA-seq UMI counts, and relative protein abundances for all samples in this study are available via the Open Targets website [https://www.opentargets.org/projects/effectorness]. Web applications: Interactive applications to visualize bulk RNA/protein expression, as well as single-cell RNA expression profiles of resting and cytokine-polarized T cells are available via the Open Targets website [https://www.opentargets.org/projects/effectorness].

## Code availability

All the codes used for processing and analyzing the data in this study were compiled into a single publicly available GitHub repository [https://github.com/eddiecg/T-cell-effectorness]. Additionally, the functions used for deriving cell state-specific proteogenomic signatures are available as an R package in GitHub [https://github.com/eddiecg/proteogenomic].
