# Supplementary material for: Single-cell transcriptomics identifies an effectorness gradient shaping the response of CD4+ T cells to cytokines
Source: Nat Commun. 2020 Apr 14;11:1801. doi: 10.1038/s41467-020-15543-y (PMC7156481; doi:10.1038/s41467-020-15543-y)
Supplement: Supplementary file 1 — Supplementary Information [file 41467_2020_15543_MOESM1_ESM.pdf]

Supplementary Information

Single-cell transcriptomics identifies an effectorness gradient shaping the response of CD4+ T cells to cytokines

Cano-Gamez E and Soskic B et al

Supplementary Figures

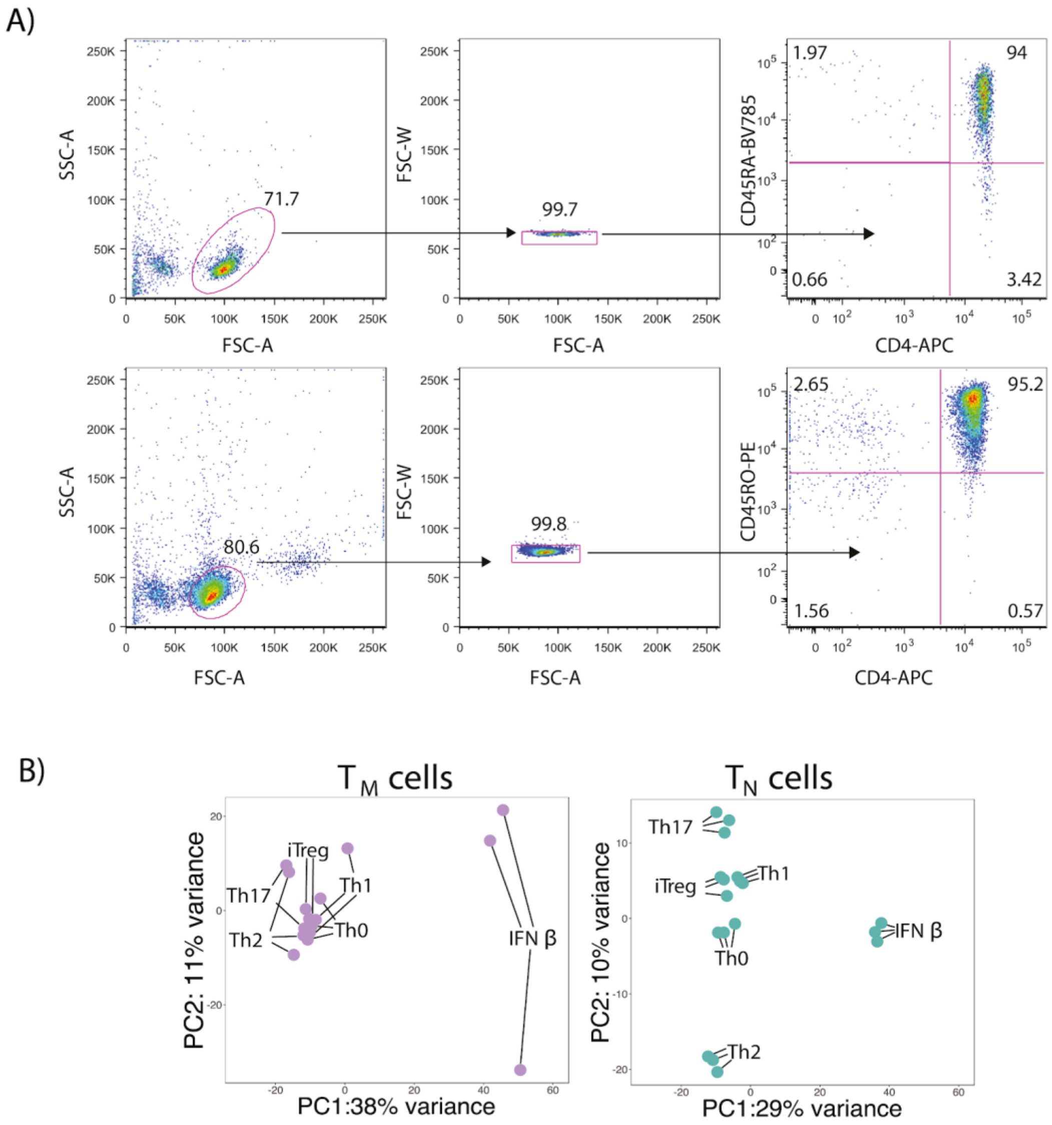

Supplementary Figure 1. Purity and quality of samples. A) Representative flow cytometry plots from three biologically independent samples. These profiles refer to the cells depicted in the schematic in Figure 1A. **B)** PCA plots from the full transcriptome of T_N and T_M cells following 16 hours of cytokine stimulations. Only stimulated cells were included in this analysis. PCA plots were derived using 21 naive and 20 memory T cells. Source data are provided as a Source Data file.

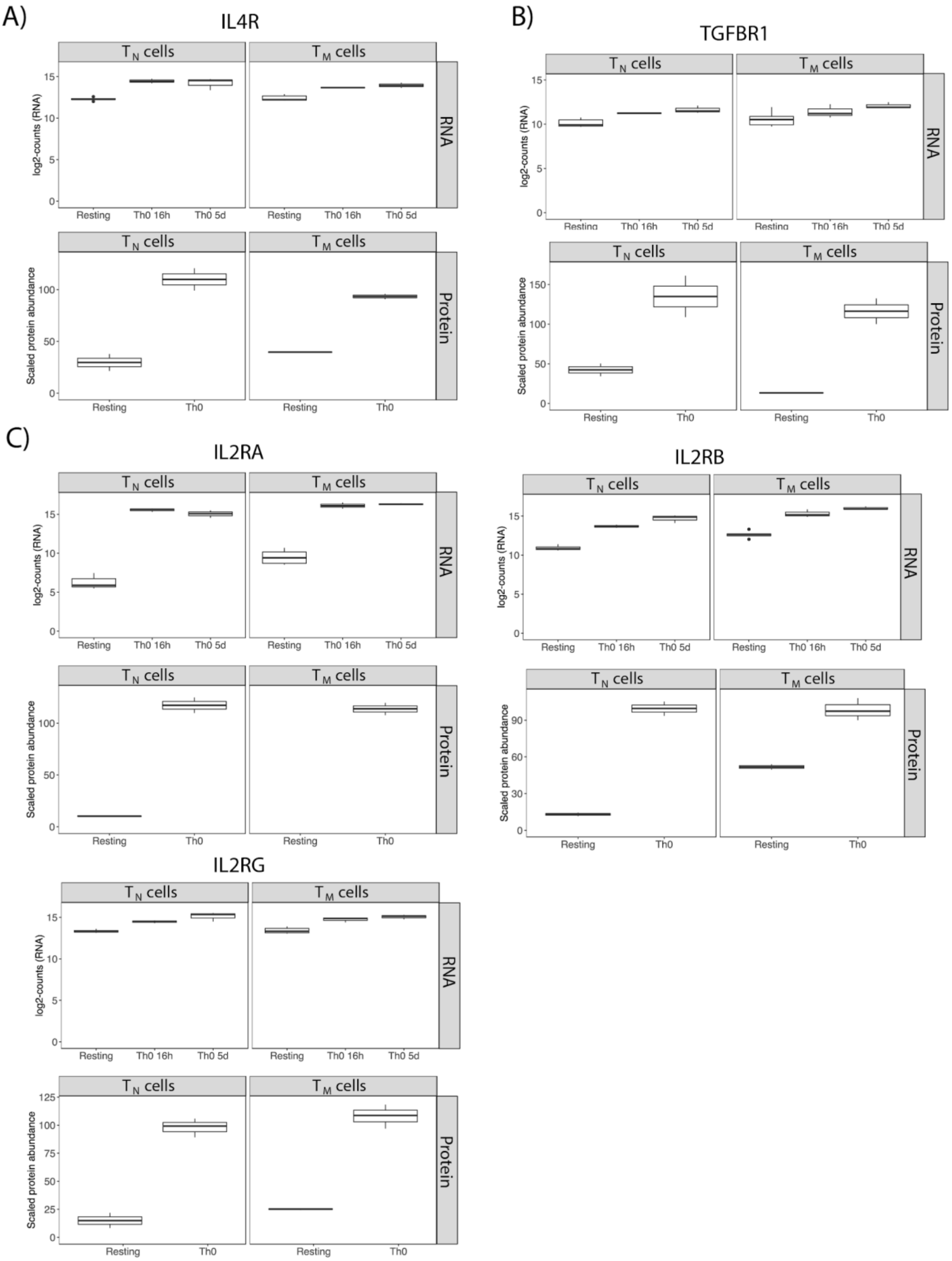

Supplementary Figure 2. Expression of receptors for cytokines with differential effects between T_N and T_M cells. RNA levels and protein abundances of cytokine receptor components in resting and stimulated T_N and T_M cells. Subunits of the **A)** IL-4, **B)** TGF-β and **C)** IL-2 receptors. Each boxplot was generated using three biologically independent samples. Boxplot centers represent median values, while the boxplot bounds represent the 25% and 75% quantiles. Boxplot whiskers represent the 25% quantile – 1.5*interquartile range (IQR) and the 75% quantile + 1.5*IQR, respectively. Source data are provided as a Source Data file.

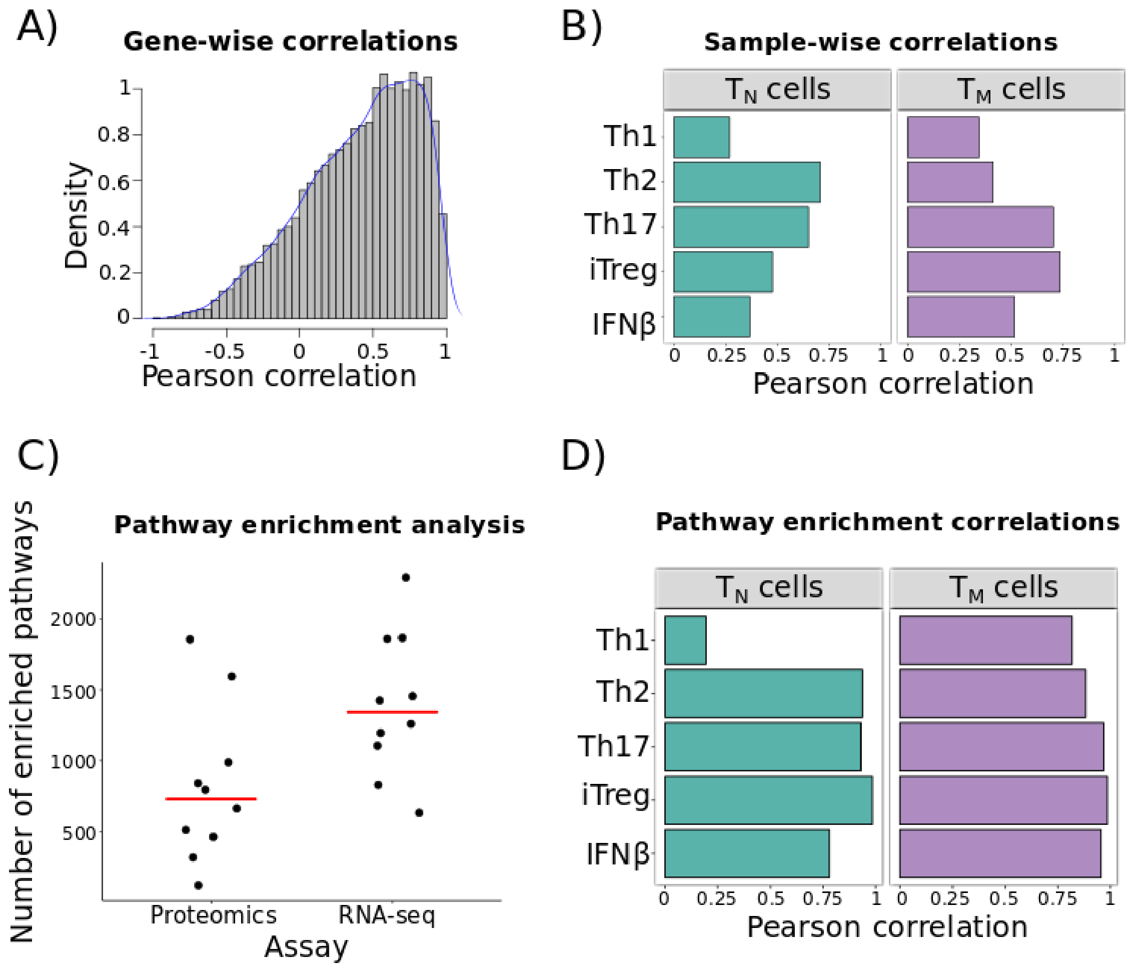

Supplementary Figure 3. Correlation between RNA and protein expression. A) Pearson correlation between RNA and protein log-fold changes for each gene across all cytokine conditions and cell types. Resting cells were excluded from this analysis. **B)** Pearson correlations between RNA and protein log-fold changes for all genes within each cytokine condition in T_N and T_M cells. **C)** Number of pathways detected as significantly enriched in differentially expressed genes and proteins from each cytokine condition. Each dot represents a cytokine condition in naive or memory T cell (N = 10). **D)** Pearson correlation between the pathway enrichment estimates derived from RNA and protein expression in each cytokine condition. Only pathways with an absolute enrichment score > 0.25 in both data sets were used for this analysis. Source data are provided as a Source Data file.

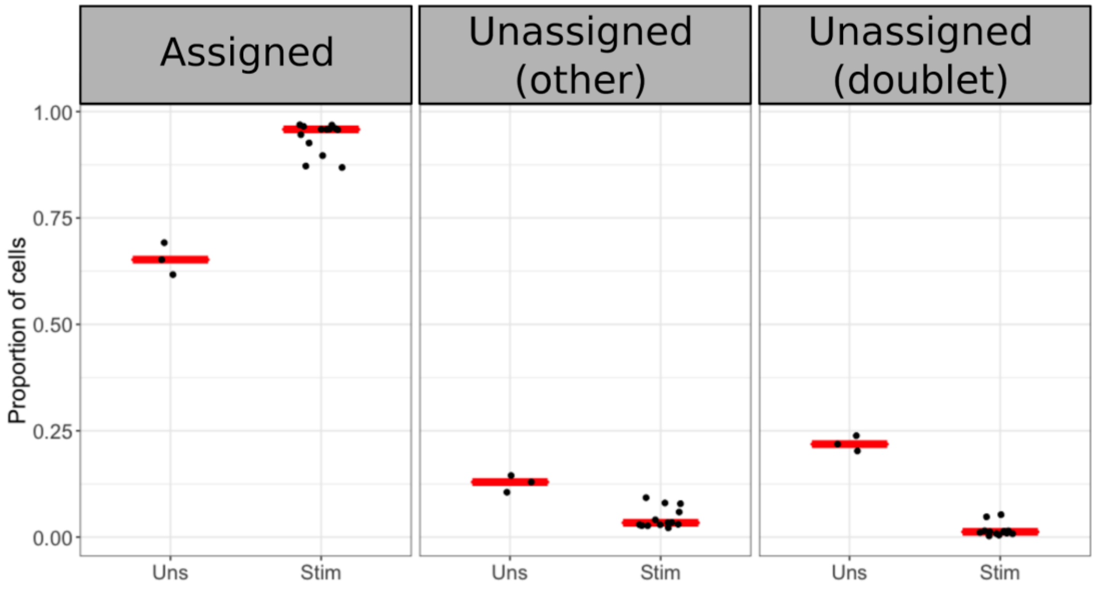

Supplementary Figure 4. **Single-cell expression of signature genes and markers in stimulated CD4+ T cells.** Percentage of cells uniquely assigned to one individual (left panel), unassigned because of low posterior probability (central panel) or containing DNA from more than one genotype (doublets, right panel). Each dot is one sample corresponding to a pool of four biologically independent samples. Source data are provided as a Source Data file.

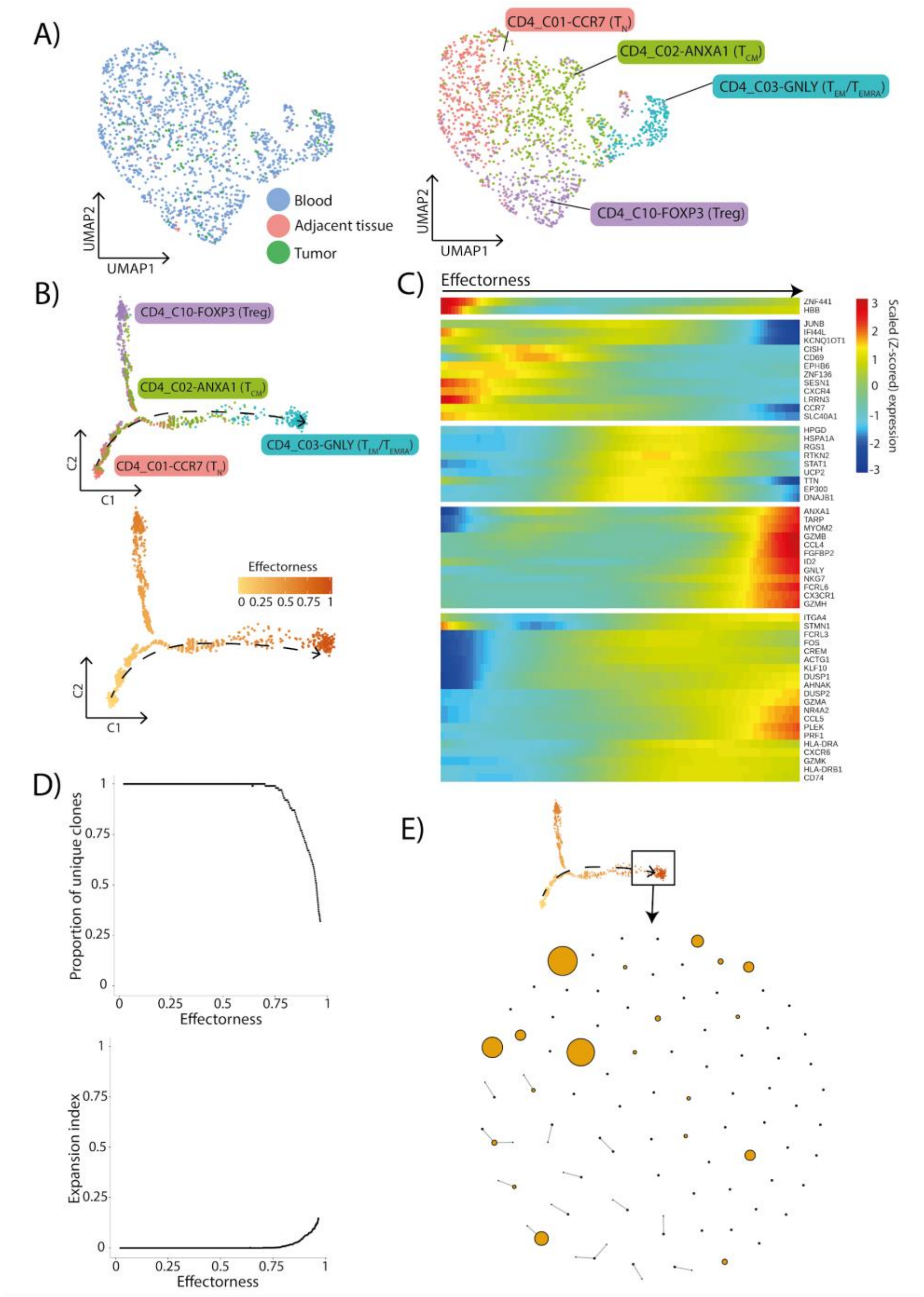

Supplementary Figure 5. **Integration of effectorness with T cell clonality** Re-analysis of public scRNA-seq data of CD4+ T cells obtained from colorectal cancer patients (**Methods**) **A)** UMAP embedding of CD4+ T cells color coded by tissue of origin (left panel) or cluster label (right panel). **B)** Cells ordered in a branched pseudotime trajectory and color coded by cluster label (top panel) or effectorness (bottom panel). **C)** Heatmap of the most variable genes along the pseudotime trajectory. Colors correspond to the scaled (Z-scored) expression of each gene in each cell. **D)** Two measurements of TCR clonotype diversity (fraction of unique clones in the left panel and expansion index in the right panel) were calculated for T cells at different ranges of effectorness using a sliding window approach (**Methods**). **E)** TCR sequences clustered by the predicted pMHC specificity of the CDR3 regions using GLIPH. Nodes represent independent TCR sequences, node size is proportional to the number of cells containing the sequence. Edges are drawn between TCR sequences predicted to recognize the same pMHC complex. Only cells with effectorness > 0.7 were included in this graph. Source data are provided as a Source Data file.

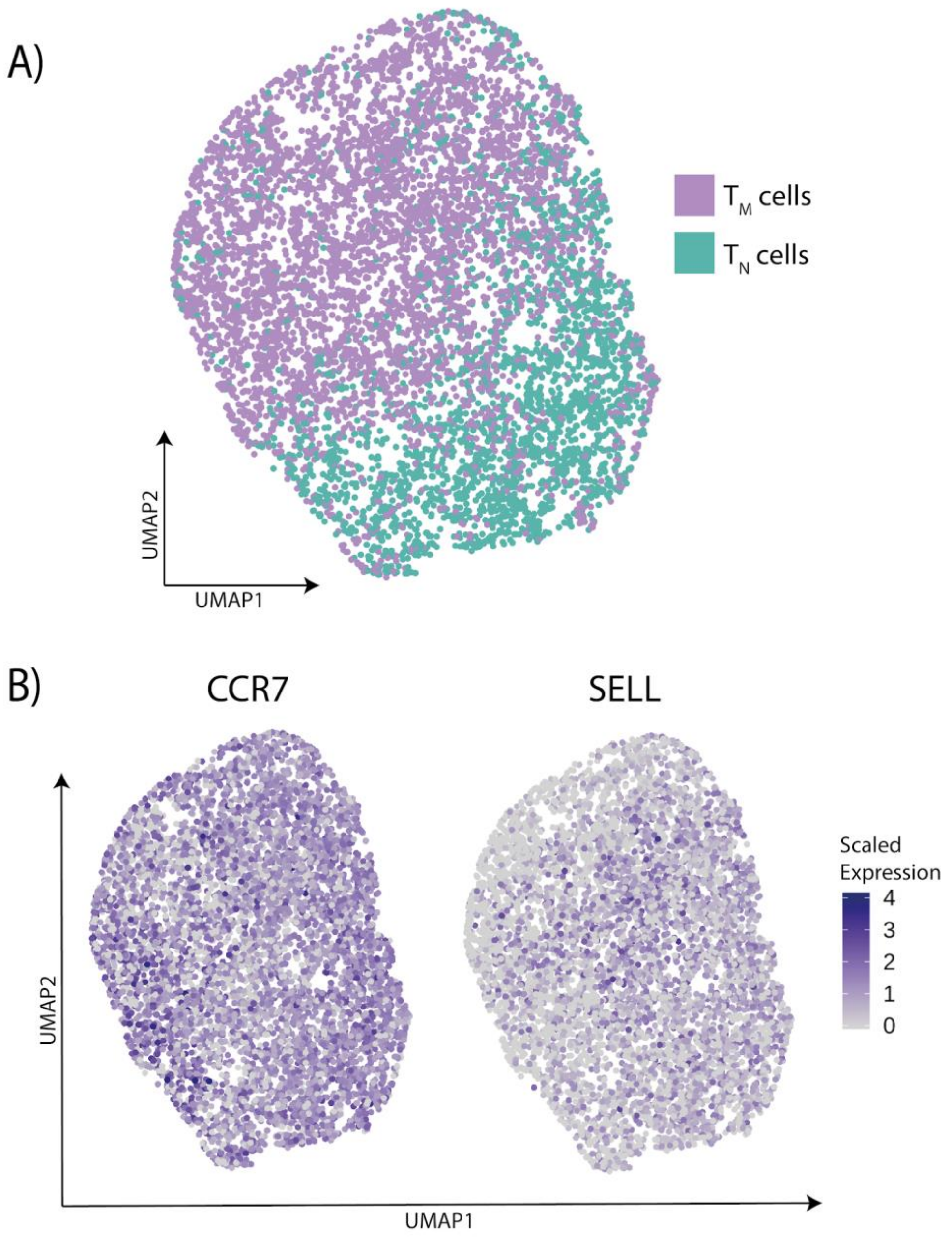

Supplementary Figure 6. **Single-cell expression of T_{CM} markers in Th0-stimulated CD4+ T cells. A)** UMAP embedding of Th0-stimulated T_N and T_M cells. Cells were color coded by either **A)** their cell type (T_N and T_M cells) or **B)** their expression levels of *SELL* (left panel) and *CCR7* (right panel). Source data are provided as a Source Data file.

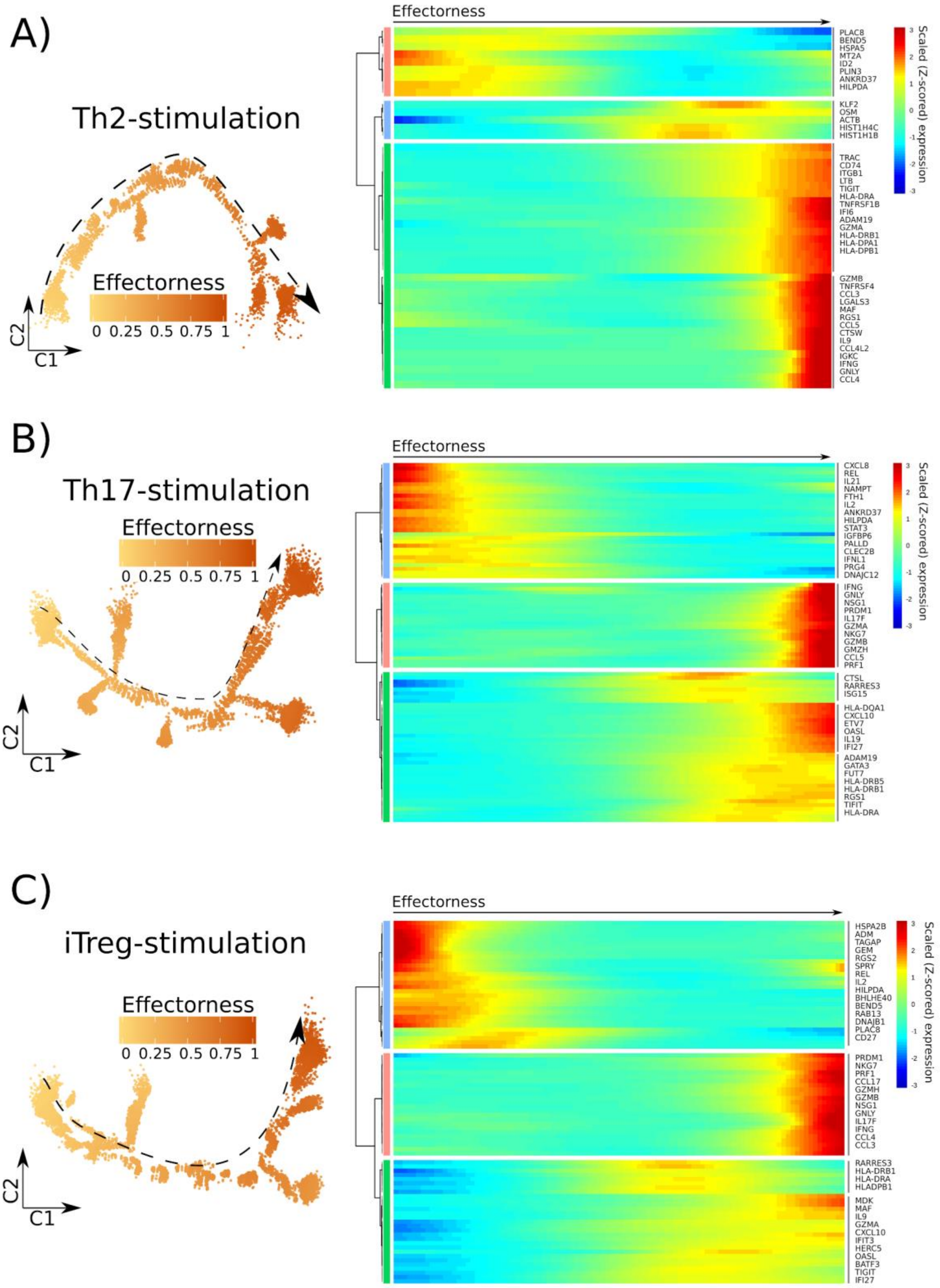

Supplementary Figure 7. **Cytokine-specific pseudotime trajectories.** T_N and T_M cells ordered in branched pseudotime trajectories. Plots are colored by pseudotime value (right panels) and accompanied by a heatmap of the most variable genes along the pseudotime trajectories (right panels). Heatmap colors correspond to the scaled (Z-scored) expression of each gene in each cell. Panels correspond to four independent trajectories from **A)** Th2, **B)** Th17 and **C)** iTreg-stimulation. Labels were added to a number of example genes. Source data are provided as a Source Data file.

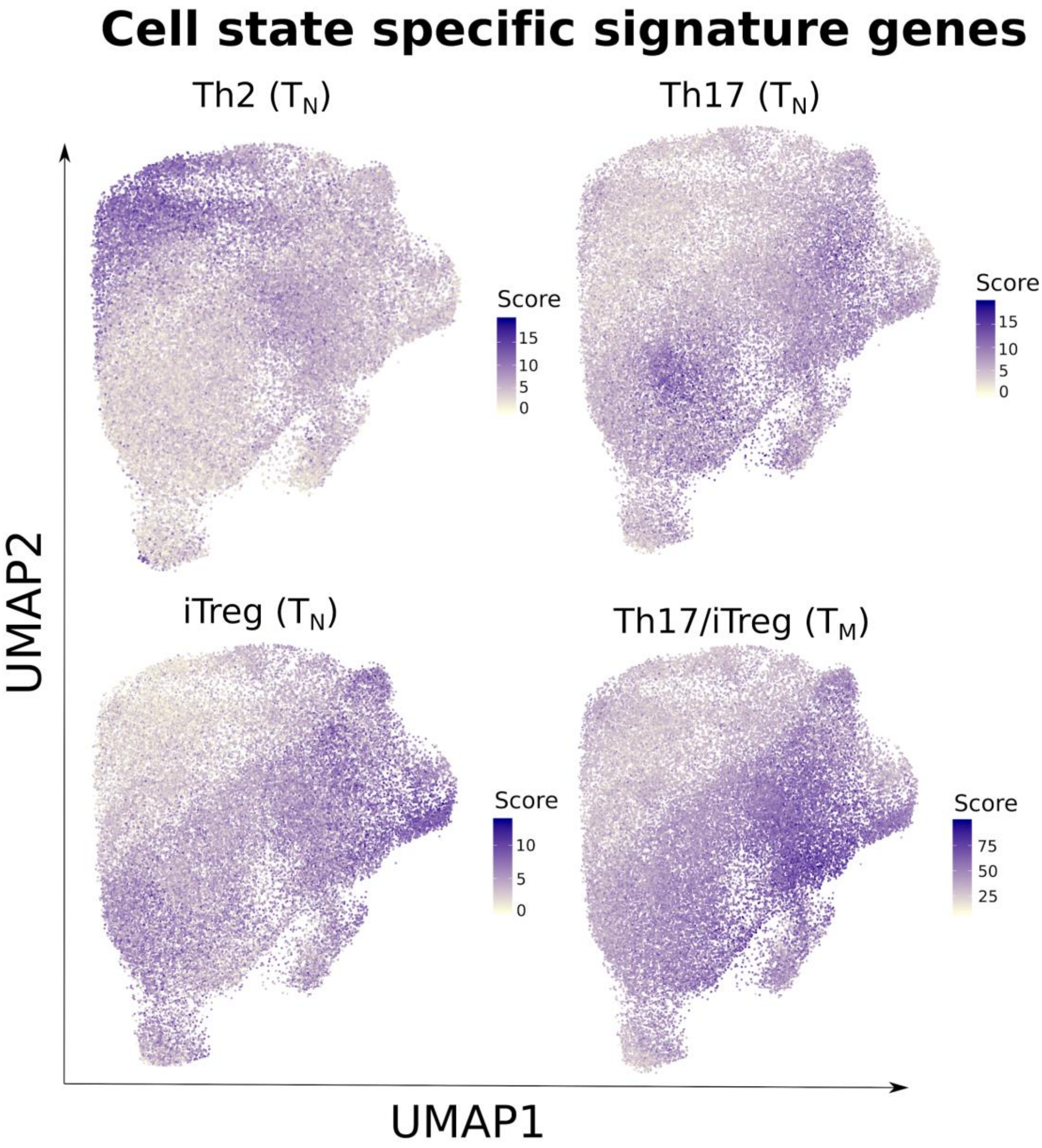

Supplementary Figure 8. **Single-cell expression of cytokine-specific signature genes in stimulated CD4+ T cells.** UMAP embedding of stimulated T_N and T_M cells from all cytokine conditions. Colors represent the average expression of all the genes in the cell state specific signatures defined from RNA-seq and proteomics data in each cell. Source data are provided as a Source Data file.

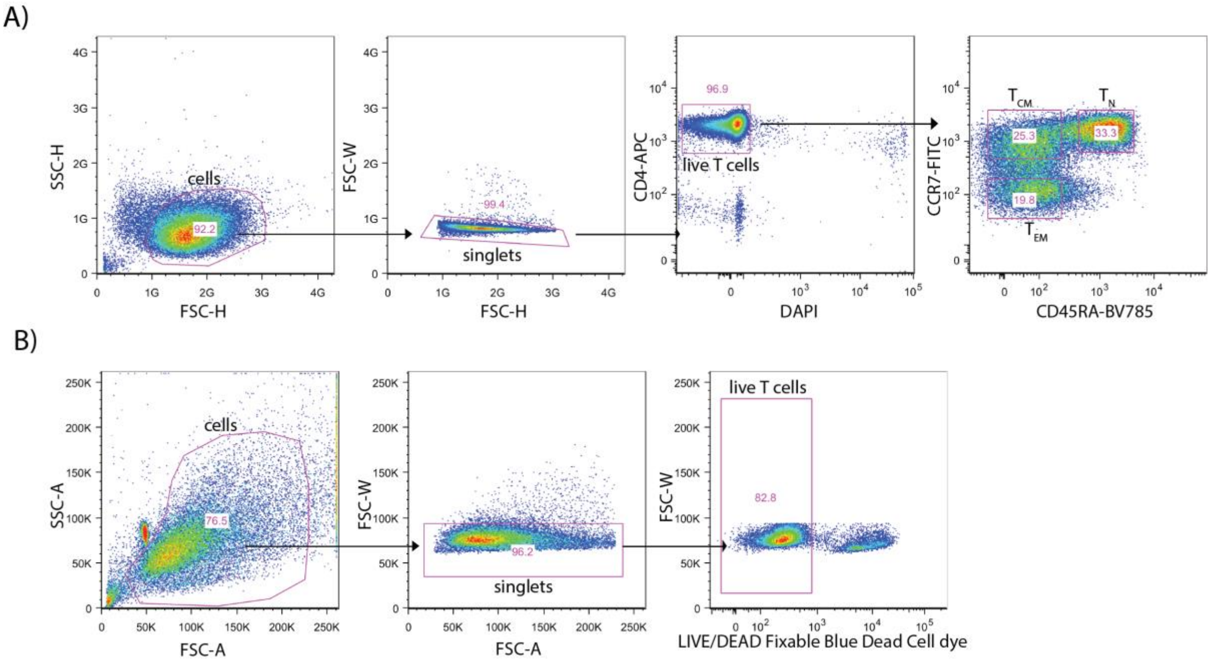

Supplementary Figure 9. **Sorting and gating strategy for cytokine staining. A)** CD4+CCR7+CD45RA+ (T_N), CD4+CCR7+CD45RA- (T_{CM}) and CD4+CCR7-CD45RA- (T_{EM}) cells were isolated from CD4+ T cells via FACS using a MoFlo XDP cell sorter. Representative flow cytometry plots of six biologically independent samples. Sorting strategy for the data presented Figure 6D. **B)** Representative flow cytometry plots of six biologically independent samples show the gating strategy for live cell isolation after restimulation. Gating strategy for the cytokine staining results presented in Figure 6D.
