# Supplementary material for: Single-cell transcriptomics identifies an effectorness gradient shaping the response of CD4+ T cells to cytokines
Source: Nat Commun. 2020 Apr 14;11:1801. doi: 10.1038/s41467-020-15543-y (PMC7156481; doi:10.1038/s41467-020-15543-y)
Supplement: Supplementary file 3 — Reporting Summary [file 41467_2020_15543_MOESM3_ESM.pdf]

nature research

Corresponding author(s): Gosia Trynka
Blagoje Soskic

Last updated by author(s): Mar 12, 2020

Reporting Summary

Nature Research wishes to improve the reproducibility of the work that we publish. This form provides structure for consistency and transparency in reporting. For further information on Nature Research policies, see Authors & Referees and the Editorial Policy Checklist.

Statistics

For all statistical analyses, confirm that the following items are present in the figure legend, table legend, main text, or Methods section.

n/a	Confirmed	
☐	☒	The exact sample size (*n*) for each experimental group/condition, given as a discrete number and unit of measurement
☐	☒	A statement on whether measurements were taken from distinct samples or whether the same sample was measured repeatedly
☐	☒	The statistical test(s) used AND whether they are one- or two-sided<br>*Only common tests should be described solely by name; describe more complex techniques in the Methods section.*
☐	☒	A description of all covariates tested
☐	☒	A description of any assumptions or corrections, such as tests of normality and adjustment for multiple comparisons
☐	☒	A full description of the statistical parameters including central tendency (e.g. means) or other basic estimates (e.g. regression coefficient) AND variation (e.g. standard deviation) or associated estimates of uncertainty (e.g. confidence intervals)
☐	☒	For null hypothesis testing, the test statistic (e.g. *F*, *t*, *r*) with confidence intervals, effect sizes, degrees of freedom and *P* value noted<br>*Give P values as exact values whenever suitable.*
☒	☐	For Bayesian analysis, information on the choice of priors and Markov chain Monte Carlo settings
☒	☐	For hierarchical and complex designs, identification of the appropriate level for tests and full reporting of outcomes
☐	☒	Estimates of effect sizes (e.g. Cohen's *d*, Pearson's *r*), indicating how they were calculated

Our web collection on statistics for biologists contains articles on many of the points above.

Software and code

Policy information about availability of computer code

Data collection	A list of common variants from exome-sequencing was collected from gnomAD (https://gnomad.broadinstitute.org/downloads, grch38 all chromosomes) and used to deconvolute single-cells by genotype.<br><br>A data set with matched scRNA-seq and paired TCR-sequences was retrieved from the study by Zhang et al. (https://www.nature.com/articles/s41586-018-0694-x). Processed counts tables were obtained from the Gene Expression Omnibus (accession number GSE108989) and metadata tables were obtained from the supplementary material.
Data analysis	Data analysis: RNA-sequencing reads were mapped to the human reference genome (GRCh38) using STAR (v2.5.3), quantified using featureCounts54 (v1.22.2) and analyzed using DESeq2 (v1.19.52) and limma (v3.35.15). Raw proteomics data were processed using the Proteome Discoverer (v2.2, Thermo Scientific) and analyzed in R using limma (v3.35.15). Pathway enrichment analysis was performed with the 1D-annotation method implemented in the Perseus software (v1.6). Single-cell RNA-sequencing data were processed and quantified using the Cell Ranger Suite (v2.2.0, 10X-Genomics) and analyzed using Seurat (v2.3.4). Pseudotime ordering was performed using Monocle (v2.12.0). Deconvolution of single-cells by genotype and assignation to individuals was done using cellSNP and Cardelino (v0.99). UniFrac distances were callculated with scUniFrac (v0.9.6). Proteogenomic signature analysis was performed in R and the respective functions are available as an R package through GitHub (https://github.com/eddiecg/proteogenomic).<br><br>Analysis of public data: Processed counts were obtained from GEO (accession number GSE108989) and analyzed using Seurat (v2.3.4). Pseudotime ordering was performed using Monocle (v2.12.0). Clustering of TCR clonotypes by specficity was performed using GLIPH (v1.0rc). The results from GLIPH were visualized using the igraph R package. The sliding-window analyses were performed using the rollapply function in the R package zoo.<br><br>All analyses were performed using publicly available R (v3.5.1). Flow cytometry data were analyzed with FlowJo (v9.9.6).

For manuscripts utilizing custom algorithms or software that are central to the research but not yet described in published literature, software must be made available to editors/reviewers. We strongly encourage code deposition in a community repository (e.g. GitHub). See the Nature Research guidelines for submitting code & software for further information.

Data

Policy information about availability of data

All manuscripts must include a data availability statement. This statement should provide the following information, where applicable:

- Accession codes, unique identifiers, or web links for publicly available datasets
- A list of figures that have associated raw data
- A description of any restrictions on data availability

Data availability:
RNA-seq raw data files have been deposited in the European Genome-Phenome Archive (EGA) under the study accession number EGAS00001003823 and the sample accession number EGAD00001005291 [https://www.ebi.ac.uk/ega/studies/EGAS00001003823]. scRNA-seq raw data files have been deposited in the European Genome-Phenome Archive (EGA) under the study accession number EGAS00001003215 and the sample accession number EGAD00001005290 [https:/www.ebi.ac.uk/ega/studies/EGAS00001003215]. Mass spectrometry raw data files have been deposited in the Proteomics Identifications Database (PRIDE) under the accession number PXD015315 [https://www.ebi.ac.uk/pride/archive/projects/PXD015315].

Count tables containing RNA-seq counts, scRNA-seq UMI counts, and relative protein abundances for all samples in this study are available via the Open Targets website [https://www.opentargets.org/projects/effectorness].

Interactive web applications to visualize bulk RNA/protein expression, as well as single-cell RNA expression profiles of resting and cytokine-polarized T cells are available via the Open Targets website [https://www.opentargets.org/projects/effectorness].

Analysis of public data:
A data set with matched scRNA-seq and paired TCR-sequences was retrieved from the study by Zhang et al. (https://www.nature.com/articles/s41586-018-0694-x). Processed counts tables were obtained from the Gene Expression Omnibus (accession number GSE108989) and metadata tables were obtained from the supplementary material.

Field-specific reporting

Please select the one below that is the best fit for your research. If you are not sure, read the appropriate sections before making your selection.

☒ Life sciences ☐ Behavioural & social sciences ☐ Ecological, evolutionary & environmental sciences

For a reference copy of the document with all sections, see nature.com/documents/nr-reporting-summary-flat.pdf

Life sciences study design

All studies must disclose on these points even when the disclosure is negative.

Sample size	We performed RNA-seq on 28 cell states and mass spectrometry on 14 cell states. Each cell state was profiled across 3 independent biological replicates. We chose this number of replicates because it enabled profiling of a large number of conditions, while retaining enough replicates per condition to infer differential expression.<br><br>We performed single-cell RNA-seq on 10 cell states. Each cell state was profiled across 4 independent biological replicates.
Data exclusions	One sample (I0766) was detected as an outlier based on principal component analysis and was removed from downstream analysis in RNAseq.
Replication	The reproducibility of sequencing and mass spectrometry data was estimated using three independent biological replicates per condition and a combination of principal component analysis and correlation analysis. Furthermore, we also sequenced three RNA-seq technical replicates. Single-cell RNA-seq was used as a validation of bulk RNA-seq results in an independent set of four biological replicates. Furthermore, we sequenced two technical replicates of the Th0 condition.
Randomization	Samples were randomized before sequencing and mass spectrometry to minimize batch effects. For smaller experiments such as FACS validations, at lest three biological replicates were included per technical run to minimize the impact of batch effects.
Blinding	No blinding was applied, as all samples were processed by the same operators

Reporting for specific materials, systems and methods

We require information from authors about some types of materials, experimental systems and methods used in many studies. Here, indicate whether each material, system or method listed is relevant to your study. If you are not sure if a list item applies to your research, read the appropriate section before selecting a response.

Materials & experimental systems

n/a	Involved in the study
☐	☒ Antibodies
☒	☐ Eukaryotic cell lines
☒	☐ Palaeontology
☒	☐ Animals and other organisms
☐	☒ Human research participants
☒	☐ Clinical data

Methods

n/a	Involved in the study
☒	☐ ChIP-seq
☐	☒ Flow cytometry
☒	☐ MRI-based neuroimaging

Antibodies

Antibodies used

Anti-human CD4, APC (BioLegend; Clone: OKT4, Catalog No: 317416, Lot no: B217365, Dilution: 1:100)
Anti-human CD45RA, Brilliant Violet 785 (BioLegend; Clone: HI100, Catalog No: 304140, Lot no: B246562, Dilution: 1:100)
Anti-human CD45RO, PE-Cyanine7 (BioLegend; Clone: UCHL1, Catalog No: 304229, Lot no: B219490, Dilution: 1:100)
Anti-CD3/anti-CD28 human T-Activator Dynabeads® (ThermoFisher, Catalog No: 111.31D, Lot no: 00670486, Concentration: 1 bead per 2 Tcells)
Anti-human IFN gamma, PE-Cyanine7 (eBioscience; Clone: 4S.B3, Catalog No: 25-7319-82, Lot no: 4328740, Dilution: 1:50)
Anti-human IL-9, PE (BD bioscience; Clone: MH9A3, Catalog No: 560814, Lot no: 9049605, Dilution: 1:50)
Anti-human CD197 (CCR7), Fitc (BD bioscience; Clone: 150503, Catalog No: 561271, Lot no: 9179069, Dilution: 1:50)
Anti-human IFN gamma, (R&D; Clone: #25718, Catalog No: MAB285-100, Lot no: KW1917101, Concentration: 1 ug/m)
Anti-human IL-4 (R&D, Clone: #34019, Catalog No: MAB204-100, Lot no: AVT0717081, Concentration: 1 ug/m)

Validation

All flow cytometry antibodies were validated by the manufacturer.
https://www.biolegend.com/ja-jp/products/apc-anti-human-cd4-antibody-3657
https://www.biolegend.com/en-us/products/brilliant-violet-785-anti-human-cd45ra-antibody-7972
https://www.biolegend.com/en-us/products/pe-cy7-anti-human-cd45ro-antibody-7760
https://www.thermofisher.com/uk/en/home/references/protocols/proteins-expression-isolation-and-analysis/t-cell-activation-and-expansion/dynabeads-human-t-activator-cd3-cd28.html
https://www.thermofisher.com/antibody/product/IFN-gamma-Antibody-clone-4S-B3-Monoclonal/25-7319-82
https://www.bdbiosciences.com/us/applications/research/t-cell-immunology/th-9-cells/intracellular-markers/cytokines-and-chemokines/human/pe-mouse-anti-human-il-9-mh9a3/p/560814
https://www.bdbiosciences.com/eu/p/561271
https://www.rndsystems.com/products/human-ifn-gamma-antibody-25718_mab285
https://www.rndsystems.com/products/human-il-4-antibody-34019_mab204

Human research participants

Policy information about studies involving human research participants

Population characteristics

Blood samples for bulk and single-cell assays were obtained from a total of 10 healthy individuals of 56.4 years of age on average (sd 12.41 years). All individuals were male and were sampled from a European population (British ancestry).

Recruitment

All blood samples were obtained via the blood and transfusion service of the NHS (UK national health service). Due to the voluntary nature of blood donations, this cohort could be affected by self-selection bias. The cohort is also slightly biased to elder individuals. However, we do not perform any inference of population parameters which could be affected by these results. Furthermore, due to the strength of our in vitro stimuli we do not expect this to impact our results.

Ethics oversight

All human biological samples were sourced ethically and their research use was in accord with the terms of the informed consents under an Institutional Review Board/Ethics Committee (IRB/EC) approved protocol (15/NW/0282).

Note that full information on the approval of the study protocol must also be provided in the manuscript.

Flow Cytometry

Plots

Confirm that:

☒ The axis labels state the marker and fluorochrome used (e.g. CD4-FITC).

☒ The axis scales are clearly visible. Include numbers along axes only for bottom left plot of group (a 'group' is an analysis of identical markers).

☒ All plots are contour plots with outliers or pseudocolor plots.

☒ A numerical value for number of cells or percentage (with statistics) is provided.

Methodology

Sample preparation

Cells were isolated from blood. Before data acquisition, cells were washed with FACS buffer (PBS buffer supplemented with 1%

Sample preparation	FCS and 1 mM EDTA) by centrifugation and stained with the respective antibodies. Reactions were incubated for 30 minutes at 4°C and resuspended in a final volume of 200 µl.
Instrument	Data was acquired using a Fortessa analyser (BD Bioscience).
Software	All data were processed with FlowJo (v9.9.6, TreeStar).
Cell population abundance	Before RNA- sequencing and mass spectrometry we assessed sample purity using flow cytometry. The populations of interest (naive and memory CD4+ T cells) were estimated to represent over 95% of the total number of cells.<br><br>When isolating memory T cell subsets, over 95% of cells were alive. Each subpopulation (naive, central memory and effector memory T cells) represented between 20% and 35% of all alive cells.
Gating strategy	We gated on FSC-A/SSC-A for cells and FSC-A/FSC-W for singlets which was proceeded by the gating for purity markers. FACS analysis was performed for purity checks, the cells that underwent sequencing were isolated using magnetic beads.<br>For naive, central and effector memory T cell isolation, we first gated on FSC-H/SSC-H and then on FSC-H/FSC-W followed by DAPI- gate for live cells. Naive T cells were defined as CD4+, CD45RA+, CCR7+. Central memory T cells were defined as CD4+, CD45RA-, CCR7+. Effector memory T cells were defined as CD4+, CD45RA-, CCR7-. For IL-9 and IFNg staining we sorted we first gated on FSC-A/SSC-A and then on FSC-A/FSC-W followed by LIVE/DEAD Fixable Blue Dead Cell dye- gate for live cells.

☒ Tick this box to confirm that a figure exemplifying the gating strategy is provided in the Supplementary Information.
