# Supplementary material for: Single-cell transcriptomics identifies an effectorness gradient shaping the response of CD4+ T cells to cytokines
Source: Nat Commun. 2020 Apr 14;11:1801. doi: 10.1038/s41467-020-15543-y (PMC7156481; doi:10.1038/s41467-020-15543-y)
Supplement: Supplementary file 4 — Description of Additional Supplementary Files [file 41467_2020_15543_MOESM4_ESM.pdf]

Description of Additional Supplementary Files

Supplementary Data 1. Cytokine concentrations and reagent information. Summary of the cell states profiled and the cell culture reagents used throughout this study.

Supplementary Data 2. Differential RNA expression results. We performed differential RNA expression analysis using a Wald test (two-sided), as implemented in DESeq2. Columns correspond to gene name, gene type, mean expression, log2-fold change, log2-fold change standard error, P value and FDR-adjusted P value.

Supplementary Data 3. Differential protein expression results. We tested for differential protein expression analysis using a two-sided moderated T-test. Columns correspond to gene name, log2-fold change, P value and FDR-adjusted P value.

Supplementary Data 4. Pathway enrichment analysis results. We performed pathway enrichment analysis in each cell state using RNA and protein information. We used the Proteus suite and the 1D-enrichment method. P values were corrected for multiple testing using FDR. Columns correspond to cell state and molecular layer of information, type of biological term, name of pathway, size of pathway, enrichment score, P value, FDR, mean, and median.

Supplementary Data 5. Cell state-specific gene signatures. We used RNA-seq and proteomics data to derive cell state specific signatures. Empirical P values were inferred using a permutation test (one-sided) and corrected for multiple testing using the FDR procedure. The columns of this table correspond to cell state the signature corresponds to, gene name, specificity score, p value and adjusted (FDR) p value.

Supplementary Data 6. Cluster markers from scRNA-seq. We used clustered cells using the Louvain algorithm and identified markers for each cluster using the Wilcoxon Rank Sum test, as implemented in Seurat. We report any gene detected as marker at FDR 0.05 and with a log2-fold change higher than 0.25. Columns correspond to cluster label, marker gene name, P value, FDR-adjusted P value, average log2-fold change, percentage of cells in cluster which express the marker and percentage of cells outside the cluster which express the marker.

Supplementary Data 7. Pseudotime-associated genes. We used Monocle to order cells in a branched pseudotime trajectory and to identify genes which significantly increase or decrease with pseudotime. P values were corrected for multiple testing and used to derive Q values. This was done separately for each cytokine condition. We report all genes with a Q value < 0.001 for an association with pseudotime. Columns correspond to gene name, P value, Q value, and total number of cells expressing the gene.

Supplementary Data 8. Effectorness-dependent genes. We modelled gene expression as a function of effectorness, cytokines, and their interaction, using a linear regression with interaction terms. Next, we identified genes significantly associated with each of these two factors. We used ANOVA (two-sided) to test which terms significantly improve model performance. P values were corrected for multiple testing using FDR.
