# Supplementary material for: Single-cell transcriptomics identifies an effectorness gradient shaping the response of CD4+ T cells to cytokines
Source: Nat Commun. 2020 Apr 14;11:1801. doi: 10.1038/s41467-020-15543-y (PMC7156481; doi:10.1038/s41467-020-15543-y)
Supplement: Supplementary file 2 — Peer Review File [file 41467_2020_15543_MOESM2_ESM.pdf]

Editorial Note: This manuscript has been previously reviewed at another journal that is not operating a transparent peer review scheme. This document only contains reviewer comments and rebuttal letters for versions considered at Nature Communications .

REVIEWERS' COMMENTS:

Reviewer #1 (Remarks to the Author):

The authors have adequately addressed my questions. As the MS focused on in vitro profiling and the biological relevance of these findings were unclear, it is more of a resource story.

Reviewer #2 (Remarks to the Author):

Given the inherent restrictions ion of their analyses the authors have provide new information that make their findings more convincing.

REVIEWERS' COMMENTS

Reviewer #1 (Remarks to the Author):

The authors have adequately addressed my questions. As the MS focused on in vitro profiling and the biological relevance of these findings were unclear, it is more of a resource story.
We thank the reviewer for their positive feedback.

Reviewer #2 (Remarks to the Author):

Given the inherent restrictions of their analyses the authors have provided new information that make their findings more convincing.
We thank the reviewer for their positive feedback.
